# Manipulation of the cell cycle by HIV-1

**DOI:** 10.1042/BST20250120

**Published:** 2026-10-06

**Authors:** Madison Bandini, Nathan M. Sherer

**Affiliations:** 1 McArdle Laboratory for Cancer Research (Department of Oncology), University of Wisconsin-Madison, Madison, WI, U.S.A.; 2 Institute for Molecular Virology, University of Wisconsin-Madison, Madison, WI, U.S.A.; 3 Carbone Cancer Center, University of Wisconsin-Madison, Madison, WI, U.S.A.

**Keywords:** cell cycle, checkpoint, HIV-1, Vif, virus, Vpr

## Abstract

Many viruses manipulate the host cell cycle to enhance viral replication and promote their evasion of host immune defenses. Human immunodeficiency virus type 1 (HIV-1) is no exception and triggers cell cycle arrest during its infection of replicating CD4^+^ T cells, acting predominantly through the activities of the viral proteins Vpr and Vif. Here, we summarize HIV-1’s effects on cell cycle regulation, focusing on the distinct mechanisms that underpin Vpr and Vif’s modulation of cell cycle checkpoints. We also discuss how these activities, when combined, may help the virus to enhance replication and promote reactivation from long-term latency—the major barrier to a cure for HIV/AIDS. A deeper understanding of how HIV-1 remodels the host cell cycle should reveal important new cell biology and may inform the development of therapeutic strategies relevant to curative interventions.

## Introduction

Cell division is central to biology and plays key roles in viral pathogenesis and antiviral immunity. The cell cycle governs cell division and is driven by mitogens and growth factors that signal temporal activation of cyclin-dependent kinases (CDKs) and is broadly divided into two major phases (Figure 1A): (i) interphase, the time between cell division events when chromosomes are duplicated and (ii) mitosis, when duplicated chromosomes are separated and segregated into two distinct daughter cells (reviewed in [1–4]). Interphase is subdivided into four subphases: Gap 0 (G0) phase when a cell is non-proliferative; Gap 1 (G1) phase when the cell emerges from G0 and prepares for DNA synthesis; Synthesis (S) phase when chromosomes are duplicated; and Gap 2 (G2) phase when cells repair DNA damage and prepare for mitotic entry. Mitosis (“M” phase) is also divided into distinct subphases, which include prophase, when duplicated chromosomes condense and form sister chromatids; prometaphase, during which the nuclear membrane dissolves and spindle microtubules attach to kinetochores on sister chromatids; metaphase, when sister chromatids align at the metaphase plate; anaphase, wherein sister chromatids are pulled apart driven by microtubule motor proteins; and telophase, wherein nuclear membranes are re-formed to protect each newly separated chromosome set. Cell division is completed when daughter cells ultimately separate through a final membrane abscission process known as cytokinesis.

**Figure 1:**
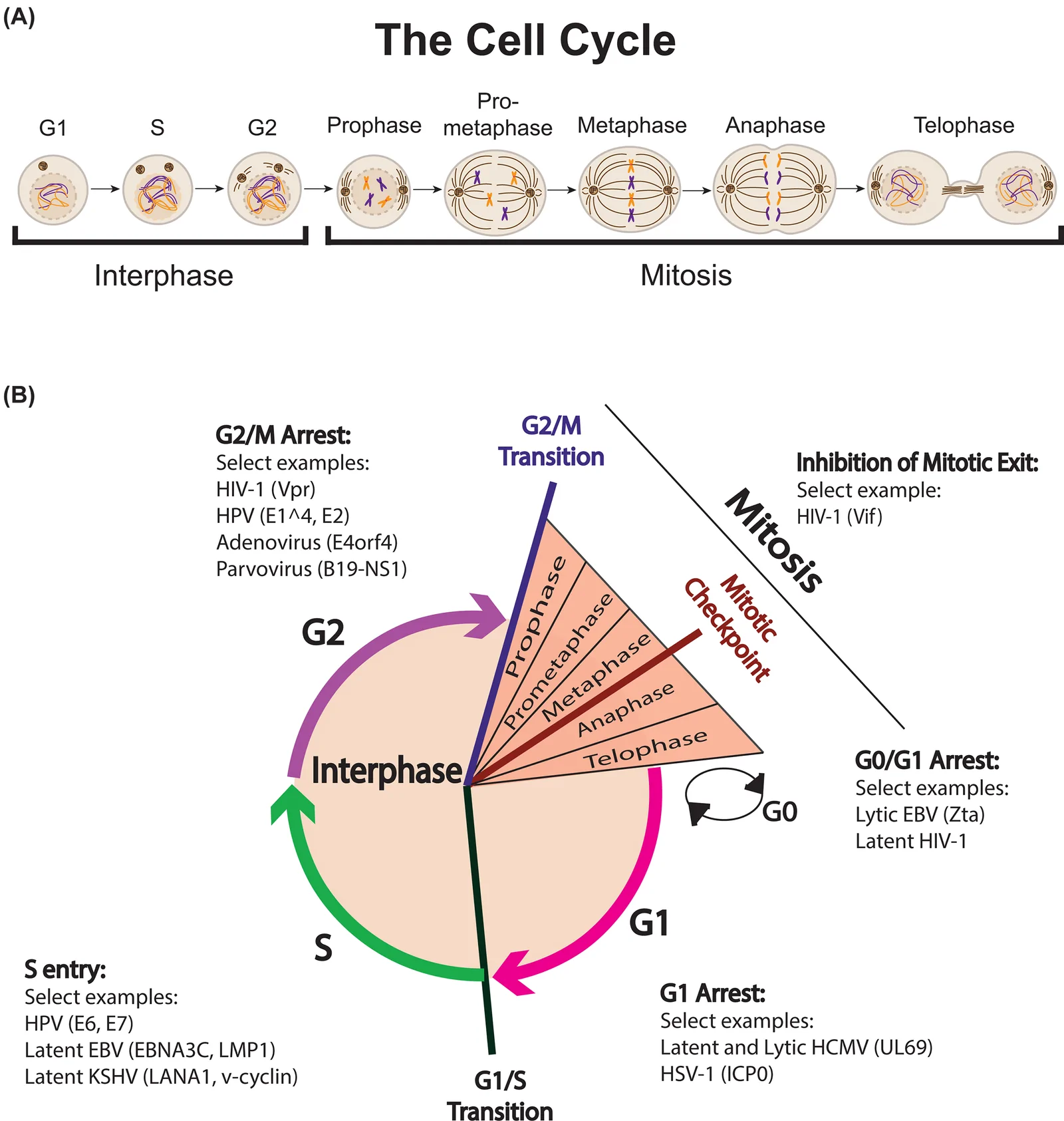
Viral modulation of the cell cycle (**A**) Schematic of the cell cycle. The cell cycle consists of interphase and mitosis. Interphase is divided into the G1, S, and G2 phases, during which cells grow, replicate their DNA, and prepare for division. Mitosis comprises prophase, prometaphase, anaphase, and telophase, followed by cytokinesis, which produces two daughter cells. (**B**) Cell cycle progression is regulated at G0/G1, G1/S, G2/M, and spindle assembly (mitotic) checkpoints. HIV-1 and other viruses manipulate host cell cycle checkpoints in ways thought to promote viral replication or immune evasion. A subset of viral effector proteins targeting cell-cycle checkpoints, and discussed in this review, are indicated; note that the figure is not intended to be comprehensive.

Progression through each phase of the cell cycle is governed by four tightly regulated checkpoints: G0/G1, G1/S, G2/M, and the mitotic checkpoint (also known as the spindle assembly checkpoint, or SAC) (Figure 1B) [3,5,6]. At each checkpoint, cells evaluate critical metabolic parameters before progressing to the next phase. The G0/G1 and G1/S checkpoints integrate signals related to cell growth, nutrient availability, mitogenic stimulation, and DNA damage, while the G2/M checkpoint monitors the success of DNA replication and genome integrity. The SAC ensures proper chromosome attachment to the mitotic spindle before anaphase, preventing chromosome mis-segregation [3,5–12]. Failure to satisfy these requirements activates checkpoint signaling pathways that can delay the cell cycle, cause senescence, or lead to programmed cell death (apoptosis).

As obligate intracellular parasites, viruses often exploit or subvert host cell cycle checkpoints to promote viral replication or evade host immune detection (select examples summarized in Figure 1B) [13–16]. The simplest means by which viruses avoid immune detection is by establishing infection in cells that cycle rarely, arrested in G0 or at the G0/G1 boundary [17]. For example, the retrovirus human immunodeficiency virus type 1 (HIV-1)—the focus of this review—infects a subset of resting memory CD4^+^ T cells to establish the viral reservoir. These cells can persist for years in a quiescent G0 state, with viral genomes hidden as integrated proviruses and held transcriptionally silenced until virus reactivation coupled to T cell activation and re-entry into the cell cycle [18–24]. Similarly, herpesviruses, including herpes simplex virus (HSV), Epstein-Barr virus (EBV), Kaposi’s sarcoma herpesvirus (KSHV), and human cytomegalovirus (HCMV), establish latent reservoirs in terminally differentiated (non-cycling) or slowly cycling cell populations, banking their large viral DNA genomes in the nucleus for years under conditions of low viral gene and antigen expression [25–30].

During active replication, many viruses arrest cells at the G0/G1, G1/S, or G2/M cell cycle transitions, exploiting these metabolically active cell growth states to express genes and produce virions. As described in detail below, HIV-1 has evolved mechanisms to subvert the G2/M transition in CD4^+^ T cells, prolonging the productive phase of infection through the coordinated activities of the viral Vif and Vpr proteins [31–37]. Herpesviruses are remarkable in that they encode much of their own machinery required for viral DNA replication, allowing them to, during their lytic (productive) growth phases, replicate genomes in cell cycle arrest states without needing to compete for the host DNA synthesis enzymes active during S phase [38–40]. Examples include EBV arresting infected cells at the G0/G1 transition through the activity of its viral Zta (BZLF1) transactivator protein that modulates the activities of the p53 and Retinoblastoma (pRb) tumor suppressor proteins [41,42]; HCMV promoting G1 arrest through the activities of its IE2 transcription factor [43], UL69 tegument protein [44,45], and RNA2.7 non-coding RNA [46], which combine to dysregulate CDK signaling [47]; and HSV type 1 halting the cell cycle at the G1/S checkpoint through the activities of its immediate-early proteins ICP0 [48–51] and ICP27 [52–54], again by targeting the functions of major host tumor suppressor proteins.

In contrast, smaller DNA viruses, including papillomaviruses and polyomaviruses, do not encode dedicated DNA replication enzymes and are dependent on the host for DNA replication and repair. Consequently, these viruses need to actively drive infected cells into S phase [55–61], with the best-characterized examples being human papillomaviruses (HPVs) that inactivate key cellular tumor suppressors through the activities of their E6 and E7 oncoproteins. E7 promotes S phase entry by targeting pRb to release the E2F transcription factors that drive cells through the G1/S checkpoint, while E6 induces degradation of p53 that might otherwise respond to DNA damage and trigger apoptosis, thereby preventing abortive infections and dampening immune detection. Persistent infection with a subset of “high-risk” HPVs (notably, HPV16 and HPV18) can lead to constitutive E6 and E7 expression due to the aberrant (non-obligatory) integration of HPV genomes into host DNA, with these effects underpinning the cause of most HPV-driven cancers [58,62–66]. Other DNA viruses, including adenoviruses, polyomaviruses, and parvoviruses, similarly converge on p53 and pRb to promote S phase and drive viral DNA synthesis [57,67–73]. Collectively, most of these small DNA viruses encode effector proteins that subsequently arrest cells at the G2/M transition by targeting CDKs or inducing the DNA damage response, e.g. HPV E1^∧^4 and E2 [74–76], parvovirus B19 NS1 [77–79], or adenovirus E4orf4 [80,81].

Importantly, EBV and KSHV also express genes during latency programs that promote cell proliferation and down-regulation of immune signaling factors, sometimes leading to cancers (e.g. EBV EBNA3C and LMP1 or KSHV LANA1 and v-cyclin) [82–86]. Similarly, HIV-1 integration into or near host genes involved in cell proliferation has recently been demonstrated to sometimes promote clonal expansion of infected cells without reactivating viral transcription, thereby expanding the viral reservoir [87–94].

Taken together, the examples above are intended to illustrate that viruses can promote or arrest cell cycle transitions in a broad variety of ways to favor viral replication or persistence, with these effects often linked to disease states that include cancers or immunodeficiencies.

## Retroviruses and the cell cycle—a brief history

HIV-1 is a retrovirus in the *lentiviridae* family and the causative agent of the acquired immunodeficiency syndrome (AIDS). AIDS has resulted in over 40 million deaths worldwide over four decades [95–97]. Like most retroviruses, HIV-1 virions encapsidate two copies of an ∼10 kb single-stranded RNA genome that is reverse transcribed into double-stranded DNA and integrated into host chromatin (Figure 2A). The HIV-1 life cycle is broadly divided into four stages (Figure 2B): (1) viral entry, initiated when the viral envelope (Env) glycoprotein binds host receptor and co-receptor proteins (Figure 2B, Step 1a), followed by membrane fusion and delivery of the capsid to the nucleus (Figure 2B, Steps 1b–1d); (2) reverse transcription and integration, in which the viral Reverse Transcriptase (RT) enzyme converts the RNA form of the genome into double-stranded DNA (Figure 2B, Step 2a) that is then integrated into the host genome by the viral Integrase (IN) enzyme (Figure 2B, Step 2b), establishing a stable provirus; (3) viral gene expression, during which host transcription machinery produces viral mRNAs and genomic RNAs (Figure 2B, Step 3a) exported from the nucleus for translation and effector functions (Figure 2B, Steps 3b–3d); and (4) virion assembly and egress, wherein the virion structural proteins Gag, Gag-Pol, and Env, and Vpr assemble at the plasma membrane to form nascent virions that bud from the cell surface (Figure 2B, Steps 4a and 4b) prior to capsid maturation mediated by the viral Protease (PR) enzyme (Figure 2B, Step 4c).

**Figure 2:**
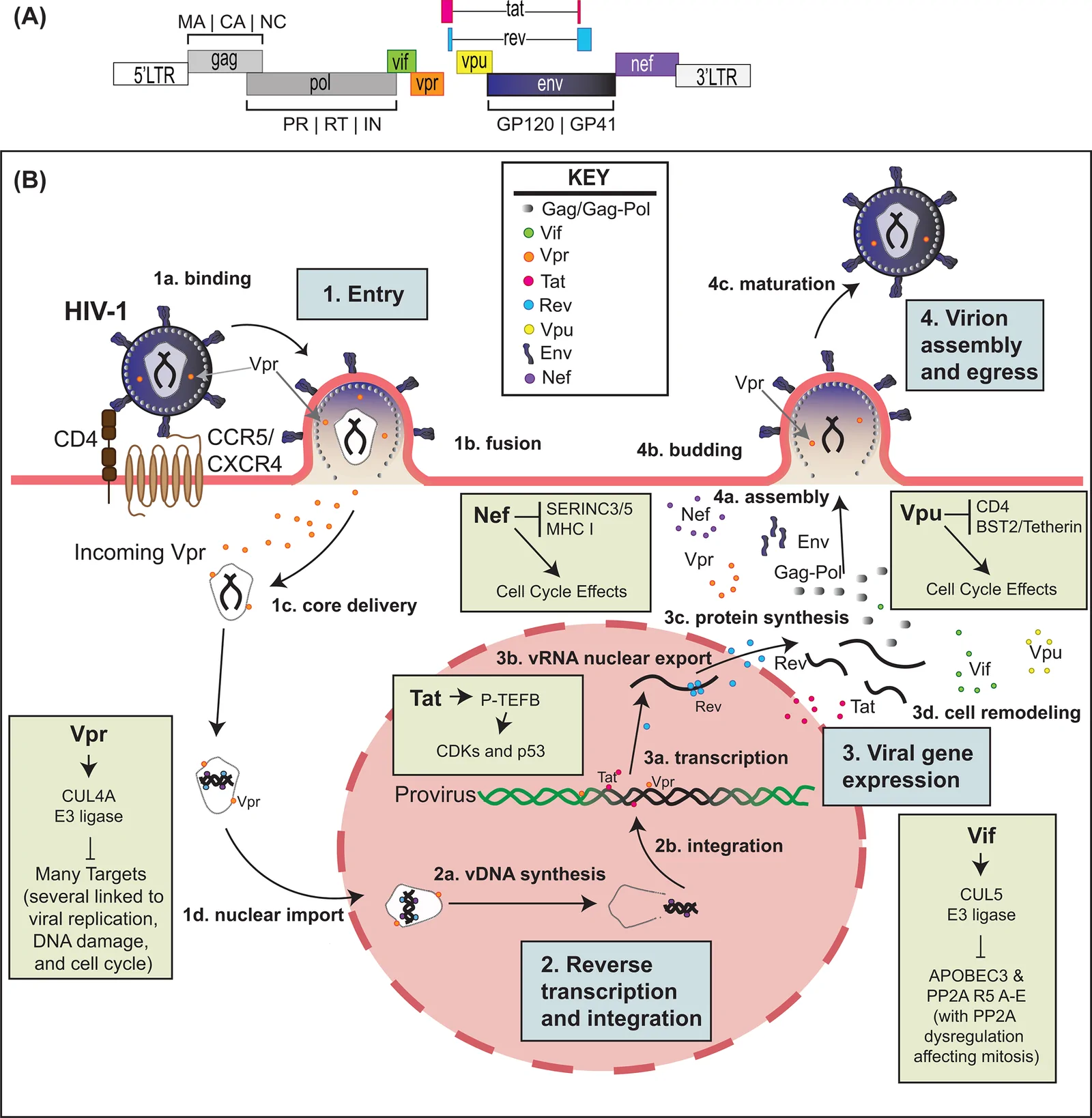
How HIV-1 affects the cell cycle (**A**) Schematic of the HIV-1 genome and encoded genes and protein subunits. In its DNA form, the viral genome is flanked by 5′ and 3′ long terminal repeats and encodes structural (*gag, gag-pol, env*), regulatory (*tat, rev*), and accessory (*vif, vpr, vpu, nef*) genes. During virion maturation, the Gag and Gag-Pol polyproteins are processed by the viral PR enzyme found in Gag-Pol to generate Gag matrix (MA), Gag capsid (CA), Gag nucleocapsid (NC), PR, RT, and IN virion proteins. Env is cleaved by host enzymes in the secretory pathway to generate gp120 and gp41 subunits. (**B**) Overview of the HIV-1 replication cycle and viral protein effects on the cell cycle broken into four stages: (1) entry, (2) reverse transcription and integration, (3) gene expression, and (4) virion assembly and egress; and the indicated substages. Canonical functions of HIV-1 proteins are indicated together with reported activities linked to cell cycle modulation.

Beyond its essential role in retroviral replication and persistence, proviral integration into host DNA can profoundly influence host cell cycle regulation with well-established links to oncogenesis. Early in the 20th century, Peyton Rous and the team of Ellermann and Bang were the first to demonstrate that certain avian cancers were caused by transmissible agents later determined to be retroviruses, marking the advent of the field of tumor virology [98,99]. Subsequent research in a variety of animal models demonstrated that retroviruses can promote cancer through at least two principal mechanisms: (i) acute transformation, in which so-called “transducing” retroviruses encode viral oncogenes that hyperactivate the cell cycle, or (ii) insertional mutagenesis, in which “non-transducing” retroviruses integrate at or near cellular proto-oncogenes or tumor suppressor genes leading to their dysregulation [100–103]. The first well-characterized transducing retrovirus, Rous sarcoma virus, was shown to drive malignant transformation through expression of the viral *v-src* oncogene, a captured version of the cellular *c-src* gene that encodes a SRC family tyrosine kinase [104–107]. Constitutive v-Src activity drives mitogenic signaling that promotes progression through the G1/S checkpoint, coupled to cytoskeletal reorganization and changes to cell morphology and motility characteristic of transformation. The discovery of v-Src led to a search for additional transducing retroviruses, leading to the discovery of major proto-oncogenes, including MYC, EGFR, RAS, and RAF [100,108–110]. In parallel, studies of non-transducing retroviruses established insertional mutagenesis as a powerful mechanism of virus-linked gene dysregulation, providing additional important insights into oncogenic signaling and aberrant cell cycle control in cancer [101,111,112].

## HIV-1 and the cell cycle

It is important to note, however, that for retroviruses cancer is typically an “accident” of viral integration and plays no obligatory role in viral replication or the spread of infection. Indeed, only a subset of retroviruses are oncogenic, and no *human* oncoretrovirus, was identified until the discovery of human T-lymphotropic virus type 1 (HTLV-1) in the 1970s [112–117]. Like HIV-1, HTLV-1 primarily infects human CD4^+^ T cells but is associated with two different major disease outcomes: HTLV-1-assciated myeopathy/topical spastic paraparesis and adult T-cell leukemia/lymphoma. HTLV-1 is thought to drive oncogenesis predominantly through the activities of its Tax protein, a transcriptional regulator that modulates cell cycle progression to promote T cell proliferation [118,119] and can promote cell changes leading to malignant transformation [118,120,121].

Unlike HTLV-1, HIV-1 does not directly cause cancer and is more likely to cause cell cycle arrest and cell death during acute infection rather than cell proliferation. Instead, HIV-1’s depletion of CD4^+^ T cells can lead to AIDS with immunodeficiency leading to increased susceptibility to opportunistic infections. Infections with oncogenic viruses such as high-risk HPV or herpesviruses, including EBV and KSHV, represent the major source of “AIDS-associated” malignancies [122,123]. Even when on antiretroviral therapy, people living with HIV remain at elevated risk for select cancers, especially those linked to high-risk HPV infection [124–128].

In addition to both activated and resting CD4^+^ T cells, HIV-1 also infects terminally differentiated CD4^+^ cells of the monocyte lineage, including macrophages, dendritic cells, and microglial cells in the brain. The virus is transmitted sexually or through blood products, and infection progresses through distinct clinical stages beginning with acute infection and high viremia followed by a prolonged asymptomatic phase during which viral replication and immune destruction continue at lower levels. Although highly active antiretroviral therapy can effectively suppress viral replication and has transformed HIV-1 infection into a manageable chronic condition, it does not eliminate latent reservoirs of infected cells, necessitating lifelong treatment [129,130]. Globally, HIV-1 remains a major public health challenge, with >40 million individuals living with HIV-1 and ∼1 million new infections annually [95].

HIV-1 targets the cell cycle in cycling T cells through multiple mechanisms. The HIV-1 genome encodes nine major proteins or polyproteins, including virion proteins Gag, Gag-Pol, and Env, with Gag-Pol representing a version of the Gag protein fused to a Pol polyprotein that encodes the viral enzymes PR, RT, and IN (Figure 2A) [103,131–134]. The genome also encodes two essential auxiliary proteins, Tat and Rev, that drive viral transcription and RNA nuclear export, respectively, and four accessory proteins (Nef, Vif, Vpr, and Vpu) that are needed for efficient replication *in vivo* because they manipulate immune signaling or modulate key cell signaling pathways [135–139]. While Vpr and Vif play the dominant roles in targeting the cell cycle (discussed in more detail below), other auxiliary or accessory proteins, including Tat, Nef, and Vpu, have been implicated in cell cycle perturbation. Tat promotes viral transcription by associating with the cellular P-TEFB kinase complex in the nucleus (Figure 2B, Step 3a) [140–142], an interaction shown in some studies to indirectly facilitate entry into S phase [143] through attenuation of Cyclin D1 expression and disruption of mitogen-activated protein kinase signaling [144]. Tat has also been reported to suppress p53-dependent transcriptional activity and checkpoint responses in a subset of studies [145–151]. Nef and Vpu perform well-established roles in immune evasion by down-regulating plasma membrane proteins that impact the efficiency of virion-cell fusion (Figure 2B, Step 1b) and virion budding/egress (Figure 2B, Step 4b) [152–161]. Nef has also been reported to influence host survival signaling pathways that intersect p53 regulation [162,163], while Vpu’s effects on SCFβ-TrCP-dependent ubiquitin signaling have also been linked to indirect effects on the cell cycle [84,146,162–167].

## HIV-1 Vpr and G2/M arrest

While Tat, Nef, and Vpu have the potential to exert measurable effects on cell cycle control through what are generally thought to be indirect mechanisms, Vpr and Vif are well established to directly mediate HIV-1–induced cell cycle modulation (with targets depicted in Figure 2B and cell effects illustrated in Figure 3).

**Figure 3:**
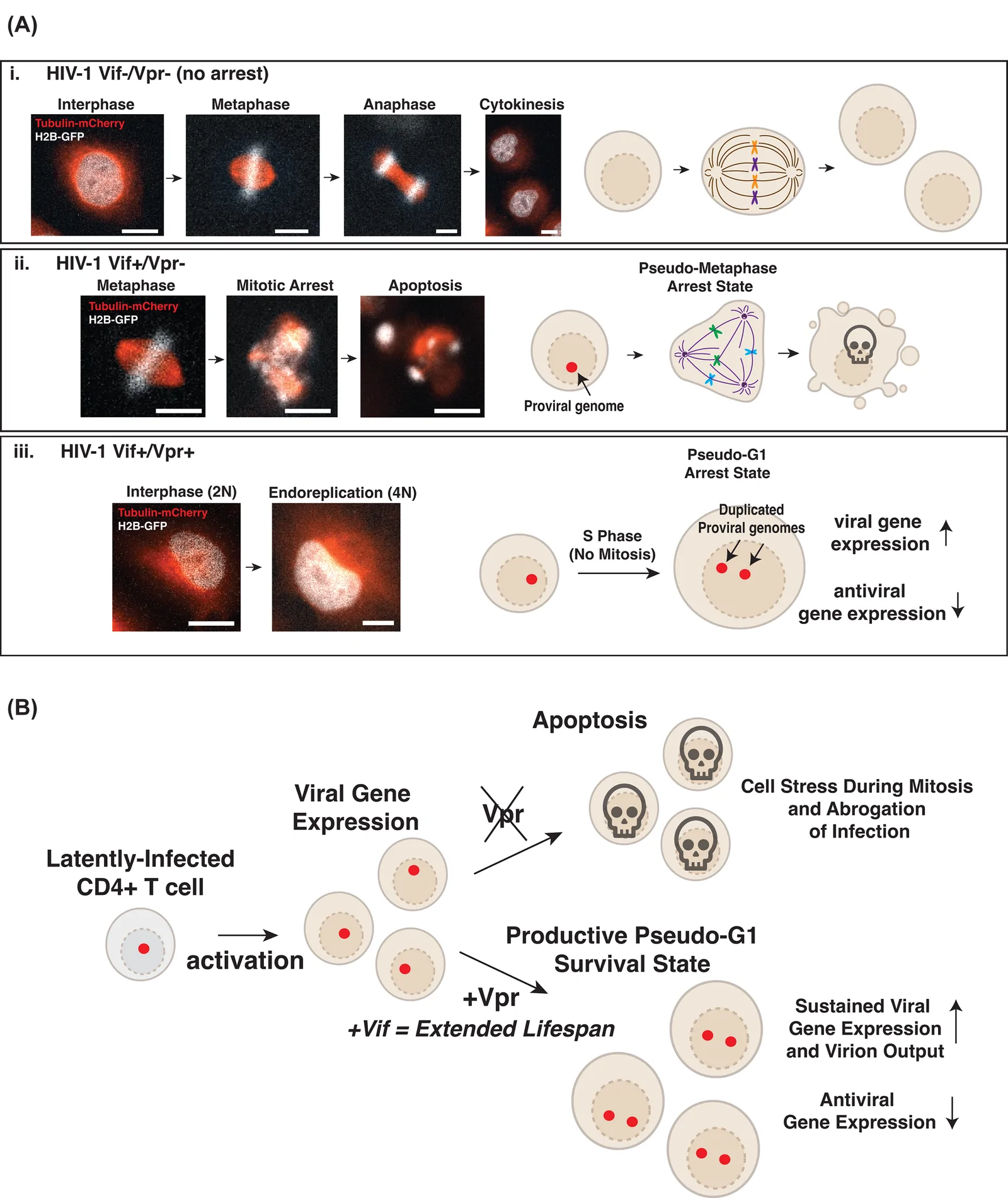
How Vif- and Vpr-mediated effects on the cell cycle can promote viral gene expression and infected cell survival (**A**) Vif and Vpr induce distinct cell cycle arrest phenotypes: pseudo-metaphase and pseudo-G1, respectively. Cell cycle reporter cells [HeLa cells constitutively expressing mCherry-Tubulin (red) to mark the mitotic spindle and H2B-GFP (white) to track chromosomes] infected with (i) Vif-Vpr−, (ii) Vif+Vpr−, or (iii) Vif+Vpr+ viruses. (i) In the absence of Vif or Vpr expression, infected cells are able to progress through a normal cell cycle, producing two daughter cells. (ii) For a virus modified to lack Vpr expression (Vif+/Vpr−), Vif causes pseudo-metaphase arrest characterized by multipolar spindles and polar chromosomes, typically resulting in cell death. (iii) For a virus expressing Vpr (Vif+/Vpr+), Vpr expression prevents mitosis and drives cells to undergo endoreplication and establishment of a pseudo-G1 state, characterized by whole genome duplication and a doubling of proviral transcription sites. (**B**) Prospective model for Vif and Vpr’s relevance to the fidelity of HIV-1 latency reversal. During activation, infected CD4^+^ T cells would be expected to re-enter the cell cycle in concert with the re-initiation of HIV-1 gene expression. We propose that the onset of Vpr expression promotes establishment of a specialized pseudo-G1/endoreplicative state that allows the virus to promote infected cell survival with enhanced viral gene expression, thereby avoiding cell suicide (apoptosis). Vif may cooperate with Vpr to extend the pseudo-G1 phase, thereby expanding the time window during which the virus can produce virions and reseed the viral reservoir. If this hypothesis is correct, then targeted inactivation of Vpr might augment the efficacy of proposed “shock-and-kill” strategies.

Vpr was the first HIV-1 accessory protein discovered to have a major effect on the cell cycle, shown in the mid-1990s [32,33,36,37] to induce morphological changes in T cells and arrest at the G2/M transition [31–33,37,168]. Vpr is a 15-kDa protein of 96 amino acids and is unique relative to the other viral accessory proteins in that it is packaged into virions during late-stage infection (Figure 2B, Step 4a) [169,170], binding to Gag for incorporation into viral particles, yielding >200 molecules of Vpr per virion [171–178]. Virion-delivered Vpr (Figure 2B, Step 1c) is sufficient to induce cell cycle arrest so that Vpr can exhibit both early (virion post-entry) and late (expressed) effects on cell function [169,171,179–182].

Mechanistically, Vpr exerts broad effects on infected cells by hijacking CUL4A-DDB1/DCAF1 E3 ubiquitin ligase complexes, resulting in the degradation or dysregulation of hundreds of cellular proteins [183–188]. Accordingly, Vpr has multivariate effects on the cell proposed to, collectively, enhance the efficiency of reverse transcription, integration, and viral gene expression in both cycling CD4^+^ T cells and non-cycling targets of infection such as macrophages [32,33,37,176,183,186,188–195]; while also suppressing cellular innate and intrinsic immune defenses [206,209–215]. DCAF1 binding is required for Vpr’s ability to induce cell cycle arrest [184,196–199], acting through a complex network of diverse effector proteins including but are not limited to MOV34, P34CDC2 (CDK1), CCDC137, SMN1, and components of the DNA repair machinery (Figure 2B) [36,37,183,188,200,201]. By disrupting DNA repair, Vpr activates DNA damage responses linked to G2/M arrest mediated by the ATM (ataxia-telangiectasia mutated) and ATR (ATM- and Rad3-related) signaling pathways [186,201–207]; with attenuation of ATR-dependent CHK1 activation shown to abrogate Vpr’s effects on G2/M [202,204,208,209]. Taken together, Vpr dramatically remodels host cells through host ubiquitin signaling upstream of DNA damage response pathways and alterations to the cell cycle.

## HIV-1 Vif causes a profound metaphase arrest

Vif is a 23-kDa protein composed of 192 amino acids, whose primary role is to target host antiviral factors in the APOBEC3 family for proteasomal degradation through recruitment of the CBF-β–ELOB/C–CUL5–RBX2 E3 ubiquitin ligase complex (Figure 2B, Step 3d) [216–223]. In doing so, Vif prevents APOBEC3 incorporation into virions, thereby protecting HIV-1 from lethal hypermutation. In addition to APOBEC3, more recent studies have identified additional Vif targets for degradation, notably the B56 family (PPP2R5A-E) of regulatory subunits of protein phosphatase 2A (PP2A) [224], fragile X mental retardation protein 1 (FMR1), and diphthamide 7 (DPH7) [225]. FMR1 interacts with HIV-1 Gag and is packaged into virions; and impacts infectivity through an unknown mechanism [226]. DPH7 plays a role in protein synthesis by modifying a core component of the translation machinery, eukaryotic elongation factor 2, but a role for DPH7 in the HIV-1 life cycle has not yet been delineated [227]. Vif was also recently implicated in modulating cellular SUMOlyation pathways, leading to alterations of host pre-mRNA splicing and gene expression [228].

Previous evidence suggested that, similar to Vpr, Vif caused G2/M cell cycle arrest, a conserved function likely to be important for the virus *in vivo* [34,35,224,229–238]. Vif-expressing cells were described with 4N DNA content and increased levels of phosphorylated checkpoint signaling proteins [229–234]. These activities required Vif’s interactions with the CUL5 E3 ubiquitin ligase complex needed for APOBEC3 degradation but were not APOBEC3-dependent [230,232]. Early models proposed that Vif interacted with Cdk9 and Brd4 to promote G1-to-S phase progression through a mechanism independent of CUL5 [239], or that Vif caused cell cycle arrest through inhibiting MDM2-mediated ubiquitination and nuclear export of p53 [35,230,240]. However, more recent studies instead implicated Vif’s targeted depletion of PPP2R5 proteins and PP2A disruption as the major source of cell cycle modulation [224,233,234,237,241]. PP2A is a heterotrimer composed of a scaffold (A), regulatory (B), and catalytic (C) subunit, with the B subunit determining substrate specificity [242]. Vif recognizes PPP2R5 subunits via electrostatic interactions and targets them for CUL5-dependent degradation, with the residues required for Vif recognition conserved across all five PPP2R5 subunits [237,241]. In some cell settings, siRNA-mediated depletion of PPP2R5 subunits has been shown to cause cell cycle arrest linked to dysregulation of phospho-signaling checkpoints [224,235,243–245].

Because prior studies studying Vif’s effects on the cell cycle were limited to flow cytometry-based measurements of DNA content at specific time points post-infection [34,233,234,237,241,246], we recently employed live cell imaging to directly visualize the effects of Vif on individual cell cycle transitions [229,231,238,247]. Using these assays, we discovered that Vif does not arrest cells at the G2/M transition as thought but instead causes cell cycle arrest during mitosis, inducing a profound metaphase arrest (normal cell cycle represented in Figure 3Ai; Vif-induced mitotic arrest in Figure 3Aii) [231]. In cell culture models, Vif-induced "pseudo-metaphase" arrest was remarkably durable, often lasting >24 h (compared with normal mitosis at ∼1 h) and characterized by reduced levels of protein phosphatases (both PP1 and PP2A) at kinetochores, destabilized forces acting on chromosomes at the mitotic spindle, and subsequent dysregulation of chromosome segregation [231]. Contrary to a prior study [35], these effects were p53-independent [231]; although the reasons for this discrepancy and if they may be linked to strain-specific differences to Vif activities [248–250] merit further study.

## Why does HIV-1 target the cell cycle, anyway?

Why Vif and Vpr pertub the cell cycle represents a longstanding question. From one perspective, triggering cell cycle arrest should represent an unfavorable strategy for a virus that banks its genome in host chromatin. On the other hand, by inducing arrest, Vif and Vpr may be acting to maintain infected, activated CD4^+^ T cells in a preferred replication state while preventing dilution of viral gene products into daughter cells. Indeed, Emerman and colleagues originally proposed that Vpr induces a prolonged G2/M arrest as a means to promote T cell “cytostaticity”, yielding a reprogrammed cell state with an extended time window for viral gene expression [33]. Consistent with a preferred cell state, subsequent studies demonstrated Vpr as enhancing viral promoter activity during the arrest phase [171,179,191,204,251–255].

Using single-cell imaging, we recently confirmed that Vpr halts the cell cycle in G2, in contrast with Vif’s targeting mitosis (Figure 3, compare 3Ai with 3Aii and 3Aiii). Remarkably, however, Vpr did not induce a “pure” G2/M arrest but instead triggered reversion to a “pseudo-G1” state without the cell proceeding to mitosis, a rare cell cycle phenotype known as “endoreplication” (Figure 3Aiii). Because HIV-1 integrates its genome directly into host chromatin, Vpr-driven endoreplication yielded polyploid cells carrying duplicated proviral genomes (Figure 3Aiii) with up-regulation of viral gene expression continuing for several days, an unexpected result considering that endoreplication typically results in transcriptional silencing [256–259].

We also observed Vpr’s effects on the cell cycle to be dominant over Vif’s, leading us to propose that, in the context of native infection, an underappreciated function of Vpr is to prevent Vif from killing cells by skipping entry into mitosis. Moreover, expression of Vif in the presence of Vpr extended time in interphase (pseudo-G1) >2-fold relative to expression of Vpr alone, suggesting that, in this context, Vif cooperates with Vpr to promote the establishment of an optimized cell survival state [229,260] (Figure 3iii). Although the mechanistic basis for Vif and Vpr’s cooperative effects remains to be elucidated, we speculate that Vif acts to enhance Vpr-mediated cytostaticity by either modulating Vpr abundance or by effecting PP2A regulatory activity [224,229,231,236,237]. It is also plausible that Vif and Vpr cooperatively target the cell cycle to suppress host immune signaling, considering that both proteins suppress diverse antiviral responses, including the DNA damage response [183,186,260–268] (Figure 3B). In this context, even virion-delivered Vpr could be relevant to immune suppression in the context of abortive infections, acting to suppress T cell proliferation through a “bystander” mechanism [169,229,272].

Vif and Vpr’s perturbation of checkpoint pathways may also play important roles in HIV-1 latency establishment by driving the selection, survival, and reprogramming of infected cells that are able to sufficiently suppress viral gene expression and cytotoxicity [269–271]. Moreover, Vpr and, to a lesser extent, Vif [239], have also been implicated in viral latency reversal, with Vpr promoting transcriptional activation in both arrested CD4^+^ T cells and non-cycling monocyte-derived macrophages and dendritic cells [191,252,269,273]. As such, further mechanistic insights into the interplay of HIV-1 latency and cell cycle control are likely of important relevance to the development of strategies aimed at reservoir elimination or durable silencing. For example, current “shock-and-kill” approaches seek to reactivate latent proviruses using latency-reversing agents (LRAs) in combination with ART to kill reservoir cells [274,275], and it was recently demonstrated that inhibitors of cell cycle regulators, including Polo-like kinases, can potentiate the efficacy of LRAs in this context [276,277]. These results are consistent with the notion that targeted inactivation of Vpr or downstream processes may yield stressed cells more susceptible to death or immune clearance (Figure 3B). However, such a strategy may be less relevant to non-cycling infected cells such as macrophages.

In conclusion, why HIV-1 targets infected cells for cell cycle arrest in the context of human infection remains to be fully elucidated. An accumulation of studies suggests that, like other viruses, Vif and Vpr’s effects on the cell cycle represent viral adaptations to reprogram cells for optimal viral replication and persistence. A more complete understanding of these dynamics may therefore reveal vulnerabilities that can be therapeutically exploited, for example, by sensitizing infected cells to apoptosis or enabling more controlled reactivation strategies that uncouple viral transcription from global T cell activation [278,279].

## Perspectives

- **Importance of the field:** Viral manipulation of the cell cycle is a fundamental strategy that enables viruses to optimize replication, evade host defenses, and establish persistent infections. Defining how HIV-1 accessory proteins rewire cell cycle pathways provides insight into mechanisms governing viral replication, latency, and infected cell survival while identifying potential therapeutic vulnerabilities.
- **Summary of current thinking:** Current evidence suggests that HIV-1 Vif and Vpr coordinately remodel the cell cycle to maximize viral fitness. Vif induces mitotic stress, whereas Vpr redirects infected cells into a pseudo-G1/endoreplicative state. Together these effects extend infected cell survival, increase viral gene expression, and promote viral replication.
- **Comment on future directions:** Future studies should determine whether disrupting HIV-1-mediated cell cycle remodeling can be exploited therapeutically during latency reversal. Inhibiting Vpr may unmask Vif-induced cytotoxicity, promoting premature death of reactivated infected cells and enhancing “shock-and-kill” strategies aimed at an HIV cure. Perturbation of checkpoint pathways may additionally influence latency establishment, proviral reactivation, and clonal survival of infected cells.

## Open Access

Open access for this article was enabled by the participation of University of Wisconsin-Madison in an all-inclusive Read & Publish agreement with Portland Press and the Biochemical Society under an agreement with the Big Ten Alliance.

## Abbreviations

AIDS: acquired immunodeficiency syndrome
CDKs: Cyclin-Dependent Kinases
DPH7: Diphthamide 7
EBV: Epstein-Barr virus
Env: envelope
FMR1: Fragile X Mental Retardation Protein 1
G0: Gap 0
G1: Gap 1
G2: Gap 2
HCMV: human cytomegalovirus
HIV-1: human immunodeficiency virus type 1
HPVs: human papillomaviruses
HSV: herpes simplex virus
HTLV-1: human T-lymphotropic virus type 1
IN: Integrase
KSHV: Kaposi’s sarcoma herpesvirus
LRAs: latency-reversing agents
M (Phase): mitosis
PP2A: Protein Phosphatase 2A
PR: Protease
RT: Reverse Transcriptase
S (Phase): synthesis phase
SAC: spindle assembly checkpoint

## References

[1] Schafer K.A. (1998) The cell cycle: a review. Vet. Pathol. 35, 461–478 9823588 10.1177/030098589803500601

[2] Wang Z. (2022) Cell cycle progression and synchronization: an overview. Methods Mol. Biol. 2579, 3–23 36045194 10.1007/978-1-0716-2736-5_1

[3] Barnum K.J. and O'Connell M.J. (2014) Cell cycle regulation by checkpoints. Methods Mol. Biol. 1170, 29–40 24906307 10.1007/978-1-4939-0888-2_2 PMC4990352

[4] Morgan D.O. (2007) The Cell Cycle: Principles of Control. New Science Press, London.

[5] Hartwell L.H. and Weinert T.A. (1989) Checkpoints: controls that ensure the order of cell cycle events. Science 246, 629–634 2683079 10.1126/science.2683079

[6] Satyanarayana A. and Kaldis P. (2009) Mammalian cell-cycle regulation: several Cdks, numerous cyclins and diverse compensatory mechanisms. Oncogene 28, 2925–2939 19561645 10.1038/onc.2009.170

[7] Kastan M.B. and Bartek J. (2004) Cell-cycle checkpoints and cancer. Nature 432, 316–323 15549093 10.1038/nature03097

[8] Shiloh Y. (2003) ATM and related protein kinases: safeguarding genome integrity. Nat. Rev. Cancer 3, 155–168 12612651 10.1038/nrc1011

[9] Musacchio A. (2015) The molecular biology of spindle assembly checkpoint signaling dynamics. Curr. Biol. 25, R1002–R1018 26485365 10.1016/j.cub.2015.08.051

[10] London N. and Biggins S. (2014) Signalling dynamics in the spindle checkpoint response. Nat. Rev. Mol. Cell Biol. 15, 736–747 25303117 10.1038/nrm3888 PMC4283840

[11] Bartek J. and Lukas J. (2007) DNA damage checkpoints: from initiation to recovery or adaptation. Curr. Opin. Cell Biol. 19, 238–245 17303408 10.1016/j.ceb.2007.02.009

[12] Roos W.P. and Kaina B. (2006) DNA damage-induced cell death by apoptosis. Trends Mol. Med. 12, 440–450 16899408 10.1016/j.molmed.2006.07.007

[13] Fan Y., Sanyal S. and Bruzzone R. (2018) Breaking bad: how viruses subvert the cell cycle. Front. Cell Infect. Microbiol. 8, 396 30510918 10.3389/fcimb.2018.00396 PMC6252338

[14] Nascimento R., Costa H. and Parkhouse R.M.E. (2012) Virus manipulation of cell cycle. Protoplasma 249, 519–528 21986922 10.1007/s00709-011-0327-9

[15] Kurapati K.R.V., Samikkannu T., Atluri V.S.R. and Nair M.P.N. (2015) Cell cycle checkpoints and pathogenesis of HIV-1 infection: a brief overview. J. Basic Clin. Physiol. Pharmacol. 26, 1–11 25046311 10.1515/jbcpp-2014-0018

[16] Chaurushiya M.S. and Weitzman M.D. (2009) Viral manipulation of DNA repair and cell cycle checkpoints. DNA Repair (Amst.) 8, 1166–1176 19473887 10.1016/j.dnarep.2009.04.016 PMC2725192

[17] Ferreira I.A.T.M., Porterfield J.Z., Gupta R.K. and Mlcochova P. (2020) Cell cycle regulation in macrophages and susceptibility to HIV-1. Viruses 12, 839 32751972 10.3390/v12080839 PMC7472357

[18] Finzi D., Blankson J., Siliciano J.D., Margolick J.B., Chadwick K., Pierson T. et al. (1999) Latent infection of CD4^+^ T cells provides a mechanism for lifelong persistence of HIV-1, even in patients on effective combination therapy. Nat. Med. 5, 512–517 10229227 10.1038/8394

[19] Persaud D., Zhou Y., Siliciano J.M. and Siliciano R.F. (2003) Latency in human immunodeficiency virus type 1 infection: no easy answers. J. Virol. 77, 1659–1665 12525599 10.1128/JVI.77.3.1659-1665.2003 PMC140995

[20] Siliciano J.D., Kajdas J., Finzi D., Quinn T.C., Chadwick K., Margolick J.B. et al. (2003) Long-term follow-up studies confirm the stability of the latent reservoir for HIV-1 in resting CD4^+^ T cells. Nat. Med. 9, 727–728 12754504 10.1038/nm880

[21] Richman D.D., Margolis D.M., Delaney M., Greene W.C., Hazuda D. and Pomerantz R.J. (2009) The challenge of finding a cure for HIV infection. Science 323, 1304–1307 19265012 10.1126/science.1165706

[22] Churchill M.J., Deeks S.G., Margolis D.M., Siliciano R.F. and Swanstrom R. (2016) HIV reservoirs: what, where and how to target them. Nat. Rev. Microbiol. 14, 55–60 26616417 10.1038/nrmicro.2015.5

[23] Chun T.W., Moir S. and Fauci A.S. (2015) HIV reservoirs as obstacles and opportunities for an HIV cure. Nat. Immunol. 16, 584–589 25990814 10.1038/ni.3152

[24] Jaafoura S., de Goër de Herve M.G., Hernandez-Vargas E.A., Hendel-Chavez H., Abdoh M., Mateo M.C. et al. (2014) Progressive contraction of the latent HIV reservoir around a core of less-differentiated CD4^+^ memory T Cells. Nat. Commun. 5, 5407 25382623 10.1038/ncomms6407 PMC4241984

[25] Sinclair J. and Sissons P. (2006) Latency and reactivation of human cytomegalovirus. J. Gen. Virol. 87, 1763–1779 16760381 10.1099/vir.0.81891-0

[26] Cohen J.I. (2020) Herpesvirus latency. J. Clin. Invest. 130, 3361–3369 32364538 10.1172/JCI136225 PMC7324166

[27] Nicoll M.P., Proença J.T. and Efstathiou S. (2012) The molecular basis of herpes simplex virus latency. FEMS Microbiol. Rev. 36, 684–705 22150699 10.1111/j.1574-6976.2011.00320.x PMC3492847

[28] Chiu Y.F. and Sugden B. (2016) Epstein-Barr virus: the path from latent to productive infection. Annu. Rev. Virol. 3, 359–372 27578440 10.1146/annurev-virology-110615-042358

[29] Speck S.H. and Ganem D. (2010) Viral latency and its regulation: lessons from the γ-herpesviruses. Cell Host Microbe 8, 100–115 20638646 10.1016/j.chom.2010.06.014 PMC2914632

[30] Broussard G. and Damania B. (2020) Regulation of KSHV latency and lytic reactivation. Viruses 12, 1034 32957532 10.3390/v12091034 PMC7551196

[31] Bartz S.R., Rogel M.E. and Emerman M. (1996) Human immunodeficiency virus type 1 cell cycle control: Vpr is cytostatic and mediates G2 accumulation by a mechanism which differs from DNA damage checkpoint control. J. Virol. 70, 2324–2331 8642659 10.1128/jvi.70.4.2324-2331.1996 PMC190074

[32] Rogel M.E., Wu L.I. and Emerman M. (1995) The human immunodeficiency virus type 1 vpr gene prevents cell proliferation during chronic infection. J. Virol. 69, 882–888 7815556 10.1128/jvi.69.2.882-888.1995 PMC188655

[33] Jowett J.B., Planelles V., Poon B., Shah N.P., Chen M.L. and Chen I.S. (1995) The human immunodeficiency virus type 1 vpr gene arrests infected T cells in the G2 + M phase of the cell cycle. J. Virol. 69, 6304–6313 7666531 10.1128/jvi.69.10.6304-6313.1995 PMC189529

[34] Sakai K., Dimas J. and Lenardo M.J. (2006) The Vif and Vpr accessory proteins independently cause HIV-1-induced T cell cytopathicity and cell cycle arrest. Proc. Natl. Acad. Sci. U.S.A. 103, 3369–3374 16492778 10.1073/pnas.0509417103 PMC1413893

[35] Izumi T., Io K., Matsui M., Shirakawa K., Shinohara M., Nagai Y. et al. (2010) HIV-1 viral infectivity factor interacts with TP53 to induce G2 cell cycle arrest and positively regulate viral replication. Proc. Natl. Acad. Sci. U.S.A. 107, 20798–20803 21071676 10.1073/pnas.1008076107 PMC2996458

[36] Re F., Braaten D., Franke E.K. and Luban J. (1995) Human immunodeficiency virus type 1 Vpr arrests the cell cycle in G2 by inhibiting the activation of p34cdc2-cyclin B. J. Virol. 69, 6859–6864 7474100 10.1128/jvi.69.11.6859-6864.1995 PMC189600

[37] He J., Choe S., Walker R., Di Marzio P., Morgan D.O. and Landau N.R. (1995) Human immunodeficiency virus type 1 viral protein R (Vpr) arrests cells in the G2 phase of the cell cycle by inhibiting p34cdc2 activity. J. Virol. 69, 6705–6711 7474080 10.1128/jvi.69.11.6705-6711.1995 PMC189580

[38] Bagga S. and Bouchard M.J. (2014) Cell cycle regulation during viral infection. In Cell Cycle Control: Mechanisms and Protocols(Noguchi E. and Gadaleta M.C., eds), pp. 165–227, Springer, New York, NY. 10.1007/978-1-4939-0888-2_10 PMC7122065 24906315

[39] Swanton C. and Jones N. (2001) Strategies in subversion: de-regulation of the mammalian cell cycle by viral gene products. Int. J. Exp. Pathol. 82, 3 11422537 10.1046/j.1365-2613.2001.00165.x PMC2517700

[40] Kalejta R.F. and Shenk T. (2002) Manipulation of the cell cycle by human cytomegalovirus. Front. Biosci. 7, D295–D306 11779699 10.2741/kalejta

[41] Cayrol C. and Flemington E. (1996) G0/G1 growth arrest mediated by a region encompassing the basic leucine zipper (bZIP) domain of the Epstein-Barr virus transactivator Zta. J. Biol. Chem. 271, 31799–31802 8943219 10.1074/jbc.271.50.31799

[42] Cayrol C. and Flemington E.K. (1996) The Epstein-Barr virus bZIP transcription factor Zta causes G0/G1 cell cycle arrest through induction of cyclin-dependent kinase inhibitors. EMBO J. 15, 2748–2759 8654372 10.1002/j.1460-2075.1996.tb00635.x PMC450210

[43] Wiebusch L. and Hagemeier C. (1999) Human cytomegalovirus 86-kilodalton IE2 protein blocks cell cycle progression in G(1). J. Virol. 73, 9274–9283 10516036 10.1128/JVI.73.11.9274-9283.1999 PMC112962

[44] Hayashi M.L., Blankenship C. and Shenk T. (2000) Human cytomegalovirus UL69 protein is required for efficient accumulation of infected cells in the G1 phase of the cell cycle. Proc. Natl. Acad. Sci. U.S.A. 97, 2692–2696 10706637 10.1073/pnas.050587597 PMC15991

[45] Lu M. and Shenk T. (1999) Human cytomegalovirus UL69 protein induces cells to accumulate in G1 phase of the cell cycle. J. Virol. 73, 676–683 9847373 10.1128/JVI.73.1.676-683.1999 PMC103874

[46] Fisher T., Mizrahi O., Tai-Schmiedel J., Nachshon A., Schwartz M., Patrick M. et al. (2026) A cytomegalovirus-encoded lncRNA blocks cell-cycle progression. Mol. Cell. 86, 1247–1259.e8 41881021 10.1016/j.molcel.2026.02.025

[47] Bresnahan W.A., Boldogh I., Thompson E.A. and Albrecht T. (1996) Human cytomegalovirus inhibits cellular DNA synthesis and arrests productively infected cells in late G1. Virology 224, 150–160 8862409 10.1006/viro.1996.0516

[48] Li H., Baskaran R., Krisky D.M., Bein K., Grandi P., Cohen J.B. et al. (2008) Chk2 is required for HSV-1 ICP0-mediated G2/M arrest and enhancement of virus growth. Virology 375, 13–23 18321553 10.1016/j.virol.2008.01.038 PMC2706573

[49] Hobbs W.E. and DeLuca N.A. (1999) Perturbation of cell cycle progression and cellular gene expression as a function of herpes simplex virus ICP0. J. Virol. 73, 8245–8255 10482575 10.1128/JVI.73.10.8245-8255.1999 PMC112842

[50] Smith M.C., Boutell C. and Davido D.J. (2011) HSV-1 ICP0: paving the way for viral replication. Future Virol. 6, 421–429 21765858 10.2217/fvl.11.24 PMC3133933

[51] Lomonte P. and Everett R.D. (1999) Herpes simplex virus type 1 immediate-early protein Vmw110 inhibits progression of cells through mitosis and from G(1) into S phase of the cell cycle. J. Virol. 73, 9456–9467 10516054 10.1128/JVI.73.11.9456-9467.1999 PMC112980

[52] Song B., Liu J.J., Yeh K.C. and Knipe D.M. (2000) Herpes simplex virus infection blocks events in the G1 phase of the cell cycle. Virology 267, 326–334 10662628 10.1006/viro.1999.0146

[53] Paladino P., Marcon E., Greenblatt J. and Frappier L. (2014) Identification of herpesvirus proteins that contribute to G1/S arrest. J. Virol. 88, 4480–4492 24501404 10.1128/JVI.00059-14 PMC3993752

[54] Hume A.J. and Kalejta R.F. (2009) Regulation of the retinoblastoma proteins by the human herpesviruses. Cell Div. 4, 1 19146698 10.1186/1747-1028-4-1 PMC2636798

[55] Flint J. and Shenk T. (1997) Viral transactivating proteins. Annu. Rev. Genet. 31, 177–212 9442894 10.1146/annurev.genet.31.1.177

[56] Moore P.S. and Chang Y. (2010) Why do viruses cause cancer? Highlights of the first century of human tumour virology Nat. Rev. Cancer 10, 878–889 21102637 10.1038/nrc2961 PMC3718018

[57] DeCaprio J.A. (2009) How the Rb tumor suppressor structure and function was revealed by the study of adenovirus and SV40. Virology 384, 274–284 19150725 10.1016/j.virol.2008.12.010

[58] Doorbar J., Quint W., Banks L., Bravo I.G., Stoler M., Broker T.R. et al. (2012) The biology and life-cycle of human papillomaviruses. Vaccine 30 Suppl 5, F55–F70 23199966 10.1016/j.vaccine.2012.06.083

[59] Münger K., Baldwin A., Edwards K.M., Hayakawa H., Nguyen C.L., Owens M. et al. (2004) Mechanisms of human papillomavirus-induced oncogenesis. J. Virol. 78, 11451–11460 15479788 10.1128/JVI.78.21.11451-11460.2004 PMC523272

[60] Jansen-Dürr P. (1996) How viral oncogenes make the cell cycle. Trends Genet. 12, 270–275 8763499 10.1016/0168-9525(96)81455-7

[61] Fehr A.R. and Yu D. (2013) Control the host cell cycle: viral regulation of the anaphase-promoting complex. J. Virol. 87, 8818–8825 23760246 10.1128/JVI.00088-13 PMC3754035

[62] Dyson N., Howley P.M., Münger K. and Harlow E. (1989) The human papilloma virus-16 E7 oncoprotein is able to bind to the retinoblastoma gene product. Science 243, 934–937 2537532 10.1126/science.2537532

[63] Scheffner M., Werness B.A., Huibregtse J.M., Levine A.J. and Howley P.M. (1990) The E6 oncoprotein encoded by human papillomavirus types 16 and 18 promotes the degradation of p53. Cell 63, 1129–1136 2175676 10.1016/0092-8674(90)90409-8

[64] Münger K., Phelps W.C., Bubb V., Howley P.M. and Schlegel R. (1989) The E6 and E7 genes of the human papillomavirus type 16 together are necessary and sufficient for transformation of primary human keratinocytes. J. Virol. 63, 4417–4421 2476573 10.1128/jvi.63.10.4417-4421.1989 PMC251060

[65] Moody C.A. and Laimins L.A. (2010) Human papillomavirus oncoproteins: pathways to transformation. Nat. Rev. Cancer 10, 550–560 20592731 10.1038/nrc2886

[66] Mesri E.A., Feitelson M.A. and Munger K. (2014) Human viral oncogenesis: a cancer hallmarks analysis. Cell Host Microbe 15, 266–282 24629334 10.1016/j.chom.2014.02.011 PMC3992243

[67] Ahuja D., Sáenz-Robles M.T. and Pipas J.M. (2005) SV40 large T antigen targets multiple cellular pathways to elicit cellular transformation. Oncogene 24, 7729–7745 16299533 10.1038/sj.onc.1209046

[68] Pipas J.M. (1992) Common and unique features of T antigens encoded by the polyomavirus group. J. Virol. 66, 3979–3985 1318392 10.1128/jvi.66.7.3979-3985.1992 PMC241200

[69] Weitzman M.D. and Ornelles D.A. (2005) Inactivating intracellular antiviral responses during adenovirus infection. Oncogene 24, 7686–7696 16299529 10.1038/sj.onc.1209063

[70] Whyte P., Buchkovich K.J., Horowitz J.M., Friend S.H., Raybuck M., Weinberg R.A. et al. (1988) Association between an oncogene and an anti-oncogene: the adenovirus E1A proteins bind to the retinoblastoma gene product. Nature 334, 124–129 2968522 10.1038/334124a0

[71] DeCaprio J.A., Ludlow J.W., Figge J., Shew J.Y., Huang C.M., Lee W.H. et al. (1988) SV40 large tumor antigen forms a specific complex with the product of the retinoblastoma susceptibility gene. Cell 54, 275–283 2839300 10.1016/0092-8674(88)90559-4

[72] Babiss L.E., Ginsberg H.S. and Darnell J.E. (1985) Adenovirus E1B proteins are required for accumulation of late viral mRNA and for effects on cellular mRNA translation and transport. Mol. Cell. Biol. 5, 2552–2558 2942759 10.1128/mcb.5.10.2552-2558.1985 PMC366989

[73] Yew P.R. and Berk A.J. (1992) Inhibition of p53 transactivation required for transformation by adenovirus early 1B protein. Nature 357, 82–85 1533443 10.1038/357082a0

[74] Davy C. and Doorbar J. (2007) G2/M cell cycle arrest in the life cycle of viruses. Virology 368, 219–226 17675127 10.1016/j.virol.2007.05.043 PMC7103309

[75] Frattini M.G., Hurst S.D., Lim H.B., Swaminathan S. and Laimins L.A. (1997) Abrogation of a mitotic checkpoint by E2 proteins from oncogenic human papillomaviruses correlates with increased turnover of the p53 tumor suppressor protein. EMBO J. 16, 318–331 9029152 10.1093/emboj/16.2.318 PMC1169638

[76] Knight G.L., Pugh A.G., Yates E., Bell I., Wilson R., Moody C.A. et al. (2011) A cyclin-binding motif in human papillomavirus type 18 (HPV18) E1∧E4 is necessary for association with CDK-cyclin complexes and G2/M cell cycle arrest of keratinocytes, but is not required for differentiation-dependent viral genome amplification or L1 capsid protein expression. Virology 412, 196–210 21276999 10.1016/j.virol.2011.01.007 PMC3722429

[77] Morita E., Tada K., Chisaka H., Asao H., Sato H., Yaegashi N. et al. (2001) Human parvovirus B19 induces cell cycle arrest at G2 phase with accumulation of mitotic cyclins. J. Virol. 75, 7555–7563 11462027 10.1128/JVI.75.16.7555-7563.2001 PMC114990

[78] Lou S., Luo Y., Cheng F., Huang Q., Shen W., Kleiboeker S. et al. (2012) Human parvovirus B19 DNA replication induces a DNA damage response that is dispensable for cell cycle arrest at phase G2/M. J. Virol. 86, 10748–10758 22837195 10.1128/JVI.01007-12 PMC3457271

[79] Xu P., Zhou Z., Xiong M., Zou W., Deng X., Ganaie S.S. et al. (2017) Parvovirus B19 NS1 protein induces cell cycle arrest at G2-phase by activating the ATR-CDC25C-CDK1 pathway. PLoS Pathog. 13, e1006266 28264028 10.1371/journal.ppat.1006266 PMC5354443

[80] Kornitzer D., Sharf R. and Kleinberger T. (2001) Adenovirus E4orf4 protein induces PP2A-dependent growth arrest in *Saccharomyces cerevisiae* and interacts with the anaphase-promoting complex/cyclosome. J. Cell Biol. 154, 331–344 11470822 10.1083/jcb.200104104 PMC2150760

[81] Li S., Brignole C., Marcellus R., Thirlwell S., Binda O., McQuoid M.J. et al. (2009) The adenovirus E4orf4 protein induces G2/M arrest and cell death by blocking protein phosphatase 2A activity regulated by the B55 subunit. J. Virol. 83, 8340–8352 19535438 10.1128/JVI.00711-09 PMC2738146

[82] Raab-Traub N. (2012) Novel mechanisms of EBV-induced oncogenesis. Curr. Opin. Virol. 2, 453–458 22858118 10.1016/j.coviro.2012.07.001 PMC4617531

[83] Young L.S. and Rickinson A.B. (2004) Epstein-Barr virus: 40 years on. Nat. Rev. Cancer 4, 757–768 15510157 10.1038/nrc1452

[84] Verma S., Ali A., Arora S. and Banerjea A.C. (2011) Inhibition of β-TrcP-dependent ubiquitination of p53 by HIV-1 Vpu promotes p53-mediated apoptosis in human T cells. Blood 117, 6600–6607 21521785 10.1182/blood-2011-01-333427

[85] Friborg J., Kong W., Hottiger M.O. and Nabel G.J. (1999) p53 inhibition by the LANA protein of KSHV protects against cell death. Nature 402, 889–894 10622254 10.1038/47266

[86] Cesarman E. (2014) Gammaherpesviruses and lymphoproliferative disorders. Annu. Rev. Pathol. 9, 349–372 24111911 10.1146/annurev-pathol-012513-104656

[87] Schröder A.R.W., Shinn P., Chen H., Berry C., Ecker J.R. and Bushman F. (2002) HIV-1 integration in the human genome favors active genes and local hotspots. Cell 110, 521–529 12202041 10.1016/S0092-8674(02)00864-4

[88] Lewinski M.K., Yamashita M., Emerman M., Ciuffi A., Marshall H., Crawford G. et al. (2006) Retroviral DNA integration: viral and cellular determinants of target-site selection. PLoS Pathog. 2, e60 16789841 10.1371/journal.ppat.0020060 PMC1480600

[89] Wagner T.A., McLaughlin S., Garg K., Cheung C.Y.K., Larsen B.B., Styrchak S. et al. (2014) HIV latency. Proliferation of cells with HIV integrated into cancer genes contributes to persistent infection. Science 345, 570–573 25011556 10.1126/science.1256304 PMC4230336

[90] Cesana D., Santoni de Sio F.R., Rudilosso L., Gallina P., Calabria A., Beretta S. et al. (2017) HIV-1-mediated insertional activation of STAT5B and BACH2 trigger viral reservoir in T regulatory cells. Nat. Commun. 8, 498 28887441 10.1038/s41467-017-00609-1 PMC5591266

[91] Maldarelli F., Wu X., Su L., Simonetti F.R., Shao W., Hill S. et al. (2014) HIV latency. Specific HIV integration sites are linked to clonal expansion and persistence of infected cells. Science 345, 179–183 24968937 10.1126/science.1254194 PMC4262401

[92] Cohn L.B., Silva I.T., Oliveira T.Y., Rosales R.A., Parrish E.H., Learn G.H. et al. (2015) HIV-1 integration landscape during latent and active infection. Cell 160, 420–432 25635456 10.1016/j.cell.2015.01.020 PMC4371550

[93] Einkauf K.B., Lee G.Q., Gao C., Sharaf R., Sun X., Hua S. et al. (2019) Intact HIV-1 proviruses accumulate at distinct chromosomal positions during prolonged antiretroviral therapy. J. Clin. Invest. 129, 988–998 30688658 10.1172/JCI124291 PMC6391088

[94] De Scheerder M.A., Vrancken B., Dellicour S., Schlub T., Lee E., Shao W. et al. (2019) HIV rebound is predominantly fueled by genetically identical viral expansions from diverse reservoirs. Cell Host Microbe 26, 347–358.e7 31471273 10.1016/j.chom.2019.08.003 PMC11021134

[95] UNAIDS (2026) Global HIV & AIDS statistics — Fact sheet | UNAIDS. [Accessed 24 June 2026]. Available from: https://www.unaids.org/en/resources/fact-sheet

[96] Gallo R.C., Salahuddin S.Z., Popovic M., Shearer G.M., Kaplan M., Haynes B.F. et al. (1984) Frequent detection and isolation of cytopathic retroviruses (HTLV-III) from patients with AIDS and at risk for AIDS. Science 224, 500–503 6200936 10.1126/science.6200936

[97] Barré-Sinoussi F., Chermann J.C., Rey F., Nugeyre M.T., Chamaret S., Gruest J. et al. (1983) Isolation of a T-lymphotropic retrovirus from a patient at risk for acquired immune deficiency syndrome (AIDS). Science 220, 868–871 6189183 10.1126/science.6189183

[98] Vogt P.K. (2026) Cancer genes: origins and directions. Viruses 18, 702 42515554 10.3390/v18070702 PMC13431497

[99] Javier R.T. and Butel J.S. (2008) The history of tumor virology. Cancer Res. 68, 7693–7706 18829521 10.1158/0008-5472.CAN-08-3301 PMC3501656

[100] Vogt P.K. (2012) Retroviral oncogenes: a historical primer. Nat. Rev. Cancer 12, 639–648 22898541 10.1038/nrc3320 PMC3428493

[101] Fan H. and Johnson C. (2011) Insertional oncogenesis by non-acute retroviruses: implications for gene therapy. Viruses 3, 398–422 21994739 10.3390/v3040398 PMC3186009

[102] Burmeister T. (2001) Oncogenic retroviruses in animals and humans. Reviews in Medical Virology 11, 369–380. 10.1002/rmv.331 11746999

[103] Coffin J.M., Hughes S.H. and Varmus H.E.(eds) (1997) Retroviruses, Cold Spring Harbor Laboratory Press, Cold Spring Harbor (NY). Available from: http://www.ncbi.nlm.nih.gov/books/NBK19376/21433340

[104] Stehelin D., Varmus H.E., Bishop J.M. and Vogt P.K. (1976) DNA related to the transforming gene(s) of avian sarcoma viruses is present in normal avian DNA. Nature 260, 170–173 176594 10.1038/260170a0

[105] Hayward W.S., Neel B.G. and Astrin S.M. (1981) Activation of a cellular onc gene by promoter insertion in ALV-induced lymphoid leukosis. Nature 290, 475–480 6261142 10.1038/290475a0

[106] Payne G.S., Bishop J.M. and Varmus H.E. (1982) Multiple arrangements of viral DNA and an activated host oncogene in bursal lymphomas. Nature 295, 209–214 6276760 10.1038/295209a0

[107] Rous P. (1911) A sarcoma of the fowl transmissible by an agent separable from the tumor cells. J. Exp. Med. 13, 397–411 19867421 10.1084/jem.13.4.397 PMC2124874

[108] Kassiotis G. (2014) Endogenous retroviruses and the development of cancer. J. Immunol. 192, 1343–1349 24511094 10.4049/jimmunol.1302972 PMC3925786

[109] Bishop J.M. (1983) Cellular oncogenes and retroviruses. Annu. Rev. Biochem. 52, 301–354 6351725 10.1146/annurev.bi.52.070183.001505

[110] Varmus H. (2017) How tumor virology evolved into cancer biology and transformed oncology. Annu. Rev. Cancer Biol. 1, 1–18 10.1146/annurev-cancerbio-050216-034315

[111] Morales-Sánchez A. and Fuentes-Pananá E.M. (2014) Human viruses and cancer. Viruses 6, 4047–4079 25341666 10.3390/v6104047 PMC4213577

[112] Blattner W.A. (1999) Human retroviruses: their role in cancer. Proc. Assoc. Am. Physicians 111, 563–572 10591085 10.1046/j.1525-1381.1999.99210.x

[113] Poiesz B.J., Ruscetti F.W., Gazdar A.F., Bunn P.A., Minna J.D. and Gallo R.C. (1980) Detection and isolation of type C retrovirus particles from fresh and cultured lymphocytes of a patient with cutaneous T-cell lymphoma. Proc. Natl. Acad. Sci. U.S.A. 77, 7415–7419 6261256 10.1073/pnas.77.12.7415 PMC350514

[114] Yoshida M., Miyoshi I. and Hinuma Y. (1982) Isolation and characterization of retrovirus from cell lines of human adult T-cell leukemia and its implication in the disease. Proc. Natl. Acad. Sci. U.S.A. 79, 2031–2035 6979048 10.1073/pnas.79.6.2031 PMC346116

[115] Kalyanaraman V.S., Sarngadharan M.G., Robert-Guroff M., Miyoshi I., Golde D. and Gallo R.C. (1982) A new subtype of human T-cell leukemia virus (HTLV-II) associated with a T-cell variant of hairy cell leukemia. Science 218, 571–573 6981847 10.1126/science.6981847

[116] Rho H.M., Poiesz B., Ruscetti F.W. and Gallo R.C. (1981) Characterization of the reverse transcriptase from a new retrovirus (HTLV) produced by a human cutaneous T-cell lymphoma cell line. Virology 112, 355–360 6166122 10.1016/0042-6822(81)90642-5

[117] Popovic M., Sarin P.S., Robert-Gurroff M., Kalyanaraman V.S., Mann D., Minowada J. et al. (1983) Isolation and transmission of human retrovirus (human T-cell leukemia virus). Science 219, 856–859 6600519 10.1126/science.6600519

[118] Currer R., Duyne R.V., Jaworski E., Guendel I., Sampey G., Das R. et al. (2013) HTLV Tax: a fascinating multifunctional co-regulator of viral and cellular pathways. Front. Microbiol. 3, 406 23226145 10.3389/fmicb.2012.00406 PMC3510432

[119] Yasunaga J.I. and Matsuoka M. (2007) Human T-cell leukemia virus type I induces adult T-cell leukemia: from clinical aspects to molecular mechanisms. Cancer Control. 14, 133–140 17387298 10.1177/107327480701400206

[120] Haoudi A., Daniels R.C., Wong E., Kupfer G. and Semmes O.J. (2003) Human T-cell leukemia virus-I tax oncoprotein functionally targets a subnuclear complex involved in cellular DNA damage-response. J. Biol. Chem. 278, 37736–37744 12842897 10.1074/jbc.M301649200

[121] Durkin S.S., Guo X., Fryrear K.A., Mihaylova V.T., Gupta S.K., Belgnaoui S.M. et al. (2008) HTLV-1 tax oncoprotein subverts the cellular DNA damage response via binding to DNA-dependent protein kinase. J. Biol. Chem. 283, 36311–36320 18957425 10.1074/jbc.M804931200 PMC2605996

[122] Fauci A.S., Pantaleo G., Stanley G. and Weissman D. (1996) Immunopathogenic mechanisms of HIV infection. Annals of Internal Medicine 124, 654–663. 10.7326/0003-4819-124-7-199604010-00006 8607594

[123] Pantaleo G., Graziosi C. and Fauci A.S. (1993) The immunopathogenesis of human immunodeficiency virus infection. N. Engl. J. Med. 328, 327–335 8093551 10.1056/NEJM199302043280508

[124] Hernández-Ramírez R.U., Shiels M.S., Dubrow R. and Engels E.A. (2017) Cancer risk in HIV-infected people in the USA from 1996 to 2012: a population-based, registry-linkage study. Lancet HIV 4, e495–e504 28803888 10.1016/S2352-3018(17)30125-X PMC5669995

[125] Haas C.B., McGee-Avila J.K., Luo Q., Pfeiffer R.M., Gershman S., Cherala S. et al. (2025) Cancer incidence and trends in US adults with HIV. JAMA Oncol. 11, 855–863 40504560 10.1001/jamaoncol.2025.1589 PMC12163716

[126] Coghill A.E., Shiels M.S., Suneja G. and Engels E.A. (2015) Elevated cancer-specific mortality among HIV-infected patients in the United States. J. Clin. Oncol. 33, 2376–2383 26077242 10.1200/JCO.2014.59.5967 PMC4500831

[127] Coghill A.E., Suneja G., Rositch A.F., Shiels M.S. and Engels E.A. (2019) HIV infection, cancer treatment regimens, and cancer outcomes among elderly adults in the United States. JAMA Oncol. 5, e191742 31369037 10.1001/jamaoncol.2019.1742 PMC6681563

[128] Jin F., Vajdic C.M., Poynten I.M., McGee-Avila J.K., Castle P.E. and Grulich A.E. (2024) Cancer risk in people living with HIV and solid organ transplant recipients: a systematic review and meta-analysis. Lancet Oncol. 25, 933–944 38936380 10.1016/S1470-2045(24)00189-X PMC11246791

[129] Chun T.W., Stuyver L., Mizell S.B., Ehler L.A., Mican J.A., Baseler M. et al. (1997) Presence of an inducible HIV-1 latent reservoir during highly active antiretroviral therapy. Proc. Natl. Acad. Sci. U.S.A. 94, 13193–13197 9371822 10.1073/pnas.94.24.13193 PMC24285

[130] Siliciano R.F. and Greene W.C. (2011) HIV latency. Cold Spring Harb. Perspect. Med. 1, a007096 22229121 10.1101/cshperspect.a007096 PMC3234450

[131] Gomez C. and Hope T.J. (2005) The ins and outs of HIV replication. Cell. Microbiol. 7, 621–626 15839891 10.1111/j.1462-5822.2005.00516.x

[132] Hu W.S. and Hughes S.H. (2012) HIV-1 reverse transcription. Cold Spring Harb. Perspect. Med. 2, a006882 23028129 10.1101/cshperspect.a006882 PMC3475395

[133] Freed E.O. (2015) HIV-1 assembly, release and maturation. Nat. Rev. Microbiol. 13, 484–496 26119571 10.1038/nrmicro3490 PMC6936268

[134] Frankel A.D. and Young J.A. (1998) HIV-1: fifteen proteins and an RNA. Annu. Rev. Biochem. 67, 1–25 9759480 10.1146/annurev.biochem.67.1.1

[135] Malim M.H. and Emerman M. (2008) HIV-1 accessory proteins—ensuring viral survival in a hostile environment. Cell Host Microbe 3, 388–398 18541215 10.1016/j.chom.2008.04.008

[136] Trono D. (1995) HIV accessory proteins: leading roles for the supporting cast. Cell 82, 189–192 7628010 10.1016/0092-8674(95)90306-2

[137] Seelamgari A., Maddukuri A., Berro R., de la Fuente C., Kehn K., Deng L. et al. (2004) Role of viral regulatory and accessory proteins in HIV-1 replication. Front. Biosci. 9, 2388–2413 15353294 10.2741/1403

[138] Anderson J.L. and Hope T.J. (2004) HIV accessory proteins and surviving the host cell. Curr. HIV/AIDS Rep. 1, 47–53 16091223 10.1007/s11904-004-0007-x

[139] Emerman M. and Malim M.H. (1998) HIV-1 regulatory/accessory genes: keys to unraveling viral and host cell biology. Science 280, 1880–1884 9632380 10.1126/science.280.5371.1880

[140] Wei P., Garber M.E., Fang S.M., Fischer W.H. and Jones K.A. (1998) A novel CDK9-associated C-type cyclin interacts directly with HIV-1 Tat and mediates its high-affinity, loop-specific binding to TAR RNA. Cell 92, 451–462 9491887 10.1016/S0092-8674(00)80939-3

[141] Zhu Y., Pe'ery T., Peng J., Ramanathan Y., Marshall N., Marshall T. et al. (1997) Transcription elongation factor P-TEFb is required for HIV-1 tat transactivation *in vitro*. Genes Dev. 11, 2622–2632 9334325 10.1101/gad.11.20.2622 PMC316609

[142] Ott M., Geyer M. and Zhou Q. (2011) The control of HIV transcription: keeping RNA polymerase II on track. Cell Host Microbe 10, 426–435 22100159 10.1016/j.chom.2011.11.002 PMC3478145

[143] Fiorelli V., Gendelman R., Samaniego F., Markham P.D. and Ensoli B. (1995) Cytokines from activated T cells induce normal endothelial cells to acquire the phenotypic and functional features of AIDS-Kaposi’s sarcoma spindle cells. J. Clin. Invest. 95, 1723–1734 7535796 10.1172/JCI117849 PMC295689

[144] Mishra M., Taneja M., Malik S., Khalique H. and Seth P. (2010) Human immunodeficiency virus type 1 Tat modulates proliferation and differentiation of human neural precursor cells: implication in NeuroAIDS. J. Neurovirol. 16, 355–367 20839920 10.3109/13550284.2010.513028

[145] Souza R.P., Gimenes F., de Abreu A.L., Rocha-Brischiliari S.C., de Carvalho M.D., Ferreira E.C. et al. (2013) Differences in the mutation of the p53 gene in exons 6 and 7 in cervical samples from HIV- and HPV-infected women. Infect. Agent Cancer 8, 38 24098975 10.1186/1750-9378-8-38 PMC3851445

[146] Liu S., Guo T., Hu J., Huang W., She P. and Wu Y. (2023) HIV-1-related factors interact with p53 to influence cellular processes. AIDS Res. Ther. 20, 66 37691100 10.1186/s12981-023-00563-7 PMC10493029

[147] Li C.J., Wang C., Friedman D.J. and Pardee A.B. (1995) Reciprocal modulations between p53 and Tat of human immunodeficiency virus type 1. Proc. Natl. Acad. Sci. U.S.A. 92, 5461–5464 7777531 10.1073/pnas.92.12.5461 PMC41714

[148] Harrod R., Nacsa J., Van Lint C., Hansen J., Karpova T., McNally J. et al. (2003) Human immunodeficiency virus type-1 Tat/co-activator acetyltransferase interactions inhibit p53Lys-320 acetylation and p53-responsive transcription. J. Biol. Chem. 278, 12310–12318 12501250 10.1074/jbc.M211167200

[149] Barillari G., Palladino C., Bacigalupo I., Leone P., Falchi M. and Ensoli B. (2016) Entrance of the Tat protein of HIV-1 into human uterine cervical carcinoma cells causes upregulation of HPV-E6 expression and a decrease in p53 protein levels. Oncol. Lett. 12, 2389–2394 27698804 10.3892/ol.2016.4921 PMC5038842

[150] Ariumi Y., Kaida A., Hatanaka M. and Shimotohno K. (2001) Functional cross-talk of HIV-1 Tat with p53 through its C-terminal domain. Biochem. Biophys. Res. Commun. 287, 556–561 11554765 10.1006/bbrc.2001.5626

[151] Yoon C.H., Kim S.Y., Byeon S.E., Jeong Y., Lee J. and Kim K.P. et al.. (2015) p53-derived host restriction of HIV-1 replication by protein kinase R-mediated Tat phosphorylation and inactivation. J. Virol. 89, 4262–4280, 25653431 10.1128/JVI.03087-14 PMC4442402

[152] Margottin F., Bour S.P., Durand H., Selig L., Benichou S., Richard V. et al. (1998) A novel human WD protein, h-beta TrCp, that interacts with HIV-1 Vpu connects CD4 to the ER degradation pathway through an F-box motif. Mol. Cell. 1, 565–574 9660940 10.1016/S1097-2765(00)80056-8

[153] Van Damme N., Goff D., Katsura C., Jorgenson R.L., Mitchell R., Johnson M.C. et al. (2008) The interferon-induced protein BST-2 restricts HIV-1 release and is downregulated from the cell surface by the viral Vpu protein. Cell Host Microbe 3, 245–252 18342597 10.1016/j.chom.2008.03.001 PMC2474773

[154] Neil S.J.D., Zang T. and Bieniasz P.D. (2008) Tetherin inhibits retrovirus release and is antagonized by HIV-1 Vpu. Nature 451, 425–430 18200009 10.1038/nature06553

[155] Sauter D. and Kirchhoff F. (2018) Multilayered and versatile inhibition of cellular antiviral factors by HIV and SIV accessory proteins. Cytokine Growth Factor Rev. 40, 3–12 29526437 10.1016/j.cytogfr.2018.02.005

[156] Swigut T., Alexander L., Morgan J., Lifson J., Mansfield K.G., Lang S. et al. (2004) Impact of Nef-mediated downregulation of major histocompatibility complex class I on immune response to simian immunodeficiency virus. J. Virol. 78, 13335–13344 15542684 10.1128/JVI.78.23.13335-13344.2004 PMC525019

[157] Arhel N.J. and Kirchhoff F. (2009) Implications of Nef: host cell interactions in viral persistence and progression to AIDS. Curr. Top. Microbiol. Immunol. 339, 147–175 20012528 10.1007/978-3-642-02175-6_8

[158] Nomaguchi M., Fujita M. and Adachi A. (2008) Role of HIV-1 Vpu protein for virus spread and pathogenesis. Microbes Infect. 10, 960–967 18672082 10.1016/j.micinf.2008.07.006

[159] Saxena S.K., Shrivastava G., Tiwari S., Swamy M.A. and Nair M.P. (2012) Modulation of HIV pathogenesis and T-cell signaling by HIV-1 Nef. Future Virol. 7, 609–620 22844345 10.2217/fvl.12.42 PMC3404840

[160] Chai Q., Li S., Collins M.K., Li R., Ahmad I., Johnson S.F. et al. (2021) HIV-1 Nef interacts with the cyclin K/CDK13 complex to antagonize SERINC5 for optimal viral infectivity. Cell Rep. 36, 109514 34380030 10.1016/j.celrep.2021.109514 PMC8385645

[161] Khan N. and Geiger J.D. (2021) Role of viral protein U (Vpu) in HIV-1 infection and pathogenesis. Viruses 13, 1466 34452331 10.3390/v13081466 PMC8402909

[162] Greenway A.L., Holloway G., McPhee D.A., Ellis P., Cornall A. and Lidman M. (2003) HIV-1 Nef control of cell signalling molecules: multiple strategies to promote virus replication. J. Biosci. 28, 323–335 12734410 10.1007/BF02970151

[163] Ali A., Farooqui S.R., Rai J., Singh J., Kumar V., Mishra R. et al. (2020) HIV-1 Nef promotes ubiquitination and proteasomal degradation of p53 tumor suppressor protein by using E6AP. Biochem. Biophys. Res. Commun. 529, 1038–1044 32819562 10.1016/j.bbrc.2020.05.188

[164] Greenway A.L., McPhee D.A., Allen K., Johnstone R., Holloway G., Mills J. et al. (2002) Human immunodeficiency virus type 1 Nef binds to tumor suppressor p53 and protects cells against p53-mediated apoptosis. J. Virol. 76, 2692–2702 11861836 10.1128/JVI.76.6.2692-2702.2002 PMC135999

[165] Collins D.R. and Collins K.L. (2014) HIV-1 accessory proteins adapt cellular adaptors to facilitate immune evasion. PLoS Pathog. 10, e1003851 24465204 10.1371/journal.ppat.1003851 PMC3900642

[166] Olszewski A., Sato K., Aron Z.D., Cohen F., Harris A., McDougall B.R. et al. (2004) Guanidine alkaloid analogs as inhibitors of HIV-1 Nef interactions with p53, actin, and p56lck. Proc. Natl. Acad. Sci. U.S.A. 101, 14079–14084 15371598 10.1073/pnas.0406040101 PMC521092

[167] Estrabaud E., Rouzic E.L., Lopez-Vergès S., Morel M., Belaïdouni N., Benarous R. et al. (2007) Regulated degradation of the HIV-1 Vpu protein through a βTrCP-independent pathway limits the release of viral particles. PLoS Pathog. 3, e104 17676996 10.1371/journal.ppat.0030104 PMC1933454

[168] Levy D.N., Fernandes L.S., Williams W.V. and Weiner D.B. (1993) Induction of cell differentiation by human immunodeficiency virus 1 vpr. Cell 72, 541–550 8440020 10.1016/0092-8674(93)90073-Y

[169] Cohen E.A., Dehni G., Sodroski J.G. and Haseltine W.A. (1990) Human immunodeficiency virus vpr product is a virion-associated regulatory protein. J. Virol. 64, 3097–3099 2139896 10.1128/jvi.64.6.3097-3099.1990 PMC249501

[170] Tungaturthi P.K., Sawaya B.E., Singh S.P., Tomkowicz B., Ayyavoo V., Khalili K. et al. (2003) Role of HIV-1 Vpr in AIDS pathogenesis: relevance and implications of intravirion, intracellular and free Vpr. Biomed. Pharmacother. 57, 20–24 12642033 10.1016/S0753-3322(02)00328-1

[171] Poon B., Grovit-Ferbas K., Stewart S.A. and Chen I.S. (1998) Cell cycle arrest by Vpr in HIV-1 virions and insensitivity to antiretroviral agents. Science 281, 266–269 9657723 10.1126/science.281.5374.266

[172] Lu Y.L., Bennett R.P., Wills J.W., Gorelick R. and Ratner L. (1995) A leucine triplet repeat sequence (LXX)4 in p6gag is important for Vpr incorporation into human immunodeficiency virus type 1 particles. J. Virol. 69, 6873–6879 7474102 10.1128/jvi.69.11.6873-6879.1995 PMC189602

[173] Mahalingam S., Khan S.A., Jabbar M.A., Monken C.E., Collman R.G. and Srinivasan A. (1995) Identification of residues in the N-terminal acidic domain of HIV-1 Vpr essential for virion incorporation. Virology 207, 297–302 7871742 10.1006/viro.1995.1081

[174] Yu X.F., Matsuda M., Essex M. and Lee T.H. (1990) Open reading frame vpr of simian immunodeficiency virus encodes a virion-associated protein. J. Virol. 64, 5688–5693 2145446 10.1128/jvi.64.11.5688-5693.1990 PMC248631

[175] Levy D.N., Refaeli Y. and Weiner D.B. (1995) Extracellular Vpr protein increases cellular permissiveness to human immunodeficiency virus replication and reactivates virus from latency. J. Virol. 69, 1243–1252 7815499 10.1128/jvi.69.2.1243-1252.1995 PMC188697

[176] Henklein P., Bruns K., Sherman M.P., Tessmer U., Licha K., Kopp J. et al. (2000) Functional and structural characterization of synthetic HIV-1 Vpr that transduces cells, localizes to the nucleus, and induces G2 cell cycle arrest. J. Biol. Chem. 275, 32016–32026 10903315 10.1074/jbc.M004044200

[177] Müller B., Tessmer U., Schubert U. and Kräusslich H.G. (2000) Human immunodeficiency virus type 1 Vpr protein is incorporated into the virion in significantly smaller amounts than gag and is phosphorylated in infected cells. J. Virol. 74, 9727–9731 11000245 10.1128/JVI.74.20.9727-9731.2000 PMC112405

[178] Wanaguru M. and Bishop K.N. (2021) HIV-1 Gag recruits oligomeric Vpr via two binding sites in p6, but both mature p6 and Vpr are rapidly lost upon target cell entry. J. Virol. 95, e0055421 34106747 10.1128/JVI.00554-21 PMC8354229

[179] Yao X.J., Mouland A.J., Subbramanian R.A., Forget J., Rougeau N., Bergeron D. et al. (1998) Vpr stimulates viral expression and induces cell killing in human immunodeficiency virus type 1-infected dividing Jurkat T cells. J. Virol. 72, 4686–4693 9573232 10.1128/JVI.72.6.4686-4693.1998 PMC109992

[180] Ogawa K., Shibata R., Kiyomasu T., Higuchi I., Kishida Y., Ishimoto A. et al. (1989) Mutational analysis of the human immunodeficiency virus vpr open reading frame. J. Virol. 63, 4110–4114 2474678 10.1128/jvi.63.9.4110-4114.1989 PMC251018

[181] Poon B. and Chen I.S.Y. (2003) Human immunodeficiency virus type 1 (HIV-1) Vpr enhances expression from unintegrated HIV-1 DNA. J. Virol. 77, 3962–3972 12634356 10.1128/JVI.77.7.3962-3972.2003 PMC150668

[182] Dupont L., Bloor S., Williamson J.C., Cuesta S.M., Shah R., Teixeira-Silva A. et al. (2021) The SMC5/6 complex compacts and silences unintegrated HIV-1 DNA and is antagonized by Vpr. Cell Host Microbe 29, 792–805.e6 33811831 10.1016/j.chom.2021.03.001 PMC8118623

[183] Laguette N., Brégnard C., Hue P., Basbous J., Yatim A., Larroque M. et al. (2014) Premature activation of the SLX4 complex by Vpr promotes G2/M arrest and escape from innate immune sensing. Cell 156, 134–145 24412650 10.1016/j.cell.2013.12.011

[184] Hrecka K., Hao C., Shun M.C., Kaur S., Swanson S.K., Florens L. et al. (2016) HIV-1 and HIV-2 exhibit divergent interactions with HLTF and UNG2 DNA repair proteins. Proc. Natl. Acad. Sci. U.S.A. 113, E3921–E3930 27335459 10.1073/pnas.1605023113 PMC4941427

[185] Lahouassa H., Blondot M.L., Chauveau L., Chougui G., Morel M., Leduc M. et al. (2016) HIV-1 Vpr degrades the HLTF DNA translocase in T cells and macrophages. Proc. Natl. Acad. Sci. U.S.A. 113, 5311–5316 27114546 10.1073/pnas.1600485113 PMC4868422

[186] Greenwood E.J.D., Williamson J.C., Sienkiewicz A., Naamati A., Matheson N.J. and Lehner P.J. (2019) Promiscuous targeting of cellular proteins by Vpr drives systems-level proteomic remodeling in HIV-1 infection. Cell Rep. 27, 1579–1596.e7 31042482 10.1016/j.celrep.2019.04.025 PMC6506760

[187] Schröfelbauer B., Hakata Y. and Landau N.R. (2007) HIV-1 Vpr function is mediated by interaction with the damage-specific DNA-binding protein DDB1. Proc. Natl. Acad. Sci. U.S.A. 104, 4130–4135 17360488 10.1073/pnas.0610167104 PMC1820720

[188] Zhang F. and Bieniasz P.D. (2020) HIV-1 Vpr induces cell cycle arrest and enhances viral gene expression by depleting CCDC137. Elife 9, e55806 32538781 10.7554/eLife.55806 PMC7295576

[189] Re F., Braaten D., Franke E.K. and Luban J. (1995) Human immunodeficiency virus type 1 Vpr arrests the cell cycle in G2 by inhibiting the activation of p34cdc2-cyclin B. J. Virol. 69, 6859–6864 7474100 10.1128/jvi.69.11.6859-6864.1995 PMC189600

[190] Zhao L.J., Wang L., Mukherjee S. and Narayan O. (1994) Biochemical mechanism of HIV-1 Vpr function. Oligomerization mediated by the N-terminal domain. J. Biol. Chem. 269, 32131–32137 7798208 10.1016/S0021-9258(18)31610-7

[191] Goh W.C., Rogel M.E., Kinsey C.M., Michael S.F., Fultz P.N., Nowak M.A. et al. (1998) HIV-1 Vpr increases viral expression by manipulation of the cell cycle: a mechanism for selection of Vpr *in vivo*. Nat. Med. 4, 65–71 9427608 10.1038/nm0198-065

[192] Li G., Park H.U., Liang D. and Zhao R.Y. (2010) Cell cycle G2/M arrest through an S phase-dependent mechanism by HIV-1 viral protein R. Retrovirology 7, 59 20609246 10.1186/1742-4690-7-59 PMC2909154

[193] Zhao R.Y., Li G. and Bukrinsky M.I. (2011) Vpr-host interactions during HIV-1 viral life cycle. J. Neuroimmune Pharmacol. 6, 216–229 21318276 10.1007/s11481-011-9261-z PMC5482210

[194] Andersen J.L., Le Rouzic E. and Planelles V. (2008) HIV-1 Vpr: mechanisms of G2 arrest and apoptosis. Exp. Mol. Pathol. 85, 2–10 18514189 10.1016/j.yexmp.2008.03.015 PMC2610539

[195] Le Rouzic E., Belaïdouni N., Estrabaud E., Morel M., Rain J.C., Transy C. et al. (2007) HIV1 Vpr arrests the cell cycle by recruiting DCAF1/VprBP, a receptor of the Cul4-DDB1 ubiquitin ligase. Cell Cycle 6, 182–188 17314515 10.4161/cc.6.2.3732

[196] DeHart J.L., Zimmerman E.S., Ardon O., Monteiro-Filho C.M.R., Argañaraz E.R. and Planelles V. (2007) HIV-1 Vpr activates the G2 checkpoint through manipulation of the ubiquitin proteasome system. Virol J. 4, 57 17559673 10.1186/1743-422X-4-57 PMC1904188

[197] Tan L., Ehrlich E. and Yu X.F. (2007) DDB1 and Cul4A are required for human immunodeficiency virus type 1 Vpr-induced G2 arrest. J. Virol. 81, 10822–10830 17626091 10.1128/JVI.01380-07 PMC2045451

[198] Wen X., Duus K.M., Friedrich T.D. and de Noronha C.M.C. (2007) The HIV1 protein Vpr acts to promote G2 cell cycle arrest by engaging a DDB1 and Cullin4A-containing ubiquitin ligase complex using VprBP/DCAF1 as an adaptor. J. Biol. Chem. 282, 27046–27057 17620334 10.1074/jbc.M703955200

[199] Belzile J.P., Duisit G., Rougeau N., Mercier J., Finzi A. and Cohen É.A. (2007) HIV-1 Vpr-mediated G2 arrest involves the DDB1-CUL4AVPRBP E3 ubiquitin ligase. PLoS Pathog. 3, e85 17630831 10.1371/journal.ppat.0030085 PMC1914068

[200] Mahalingam S., Ayyavoo V., Patel M., Kieber-Emmons T., Kao G.D., Muschel R.J. et al. (1998) HIV-1 Vpr interacts with a human 34-kDa mov34 homologue, a cellular factor linked to the G2/M phase transition of the mammalian cell cycle. Proc. Natl. Acad. Sci. U.S.A. 95, 3419–3424 9520381 10.1073/pnas.95.7.3419 PMC19851

[201] Nisson K.A., Patel R.S., Delgado Y., Bouhaddou M., Etienne L. and Fregoso O.I. (2025) HIV Vpr activates a nucleolar-specific ATR pathway to degrade the nucleolar stress sensor CCDC137. Nucleic. Acids Res. 53, gkaf531 40530693 10.1093/nar/gkaf531 PMC12203918

[202] Roshal M., Kim B., Zhu Y., Nghiem P. and Planelles V. (2003) Activation of the ATR-mediated DNA damage response by the HIV-1 viral protein R. J. Biol. Chem. 278, 25879–25886 12738771 10.1074/jbc.M303948200

[203] Zimmerman E.S., Chen J., Andersen J.L., Ardon O., Dehart J.L., Blackett J. et al. (2004) Human immunodeficiency virus type 1 Vpr-mediated G2 arrest requires Rad17 and Hus1 and induces nuclear BRCA1 and gamma-H2AX focus formation. Mol. Cell. Biol. 24, 9286–9294 15485898 10.1128/MCB.24.21.9286-9294.2004 PMC522272

[204] Zimmerman E.S., Sherman M.P., Blackett J.L., Neidleman J.A., Kreis C., Mundt P. et al. (2006) Human immunodeficiency virus type 1 Vpr induces DNA replication stress *in vitro* and *in vivo*. J. Virol. 80, 10407–10418 16956949 10.1128/JVI.01212-06 PMC1641771

[205] Fregoso O.I. and Emerman M. (2016) Activation of the DNA damage response is a conserved function of HIV-1 and HIV-2 Vpr that is independent of SLX4 recruitment. mBio 7, e01433–16 27624129 10.1128/mBio.01433-16 PMC5021806

[206] Sandoval C., Nisson K. and Fregoso O.I. (2024) HIV-1 Vpr-induced DNA damage activates NF-κB through ATM-NEMO independent of cell cycle arrest. mBio 15, e0024024 39269169 10.1128/mbio.00240-24 PMC11481869

[207] Li D., Lopez A., Sandoval C., Nichols Doyle R. and Fregoso O.I. (2020) HIV Vpr modulates the host DNA damage response at two independent steps to damage DNA and repress double-strand DNA break repair. mBio 11, e00940–20 32753492 10.1128/mBio.00940-20 PMC7407082

[208] Romani B. and Engelbrecht S. (2009) Human immunodeficiency virus type 1 Vpr: functions and molecular interactions. J. Gen. Virol. 90, 1795–1805 19458171 10.1099/vir.0.011726-0

[209] Guenzel C.A., Hérate C. and Benichou S. (2014) HIV-1 Vpr-a still “enigmatic multitasker”. Front. Microbiol. 5, 127 24744753 10.3389/fmicb.2014.00127 PMC3978352

[210] Wong H.T., Luperchio A.M., Riley S. and Salamango D.J. (2023) Inhibition of ATM-directed antiviral responses by HIV-1 Vif. PLoS Pathog. 19, e1011634 37669285 10.1371/journal.ppat.1011634 PMC10503699

[211] Schwefel D., Groom H.C.T., Boucherit V.C., Christodoulou E., Walker P.A., Stoye J.P. et al. (2014) Structural basis of lentiviral subversion of a cellular protein degradation pathway. Nature 505, 234–238 24336198 10.1038/nature12815 PMC3886899

[212] Brégnard C., Benkirane M. and Laguette N. (2014) DNA damage repair machinery and HIV escape from innate immune sensing. Front. Microbiol. 5, 176 24795708 10.3389/fmicb.2014.00176 PMC4001025

[213] Khan H., Sumner R.P., Rasaiyaah J., Tan C.P., Rodriguez-Plata M.T., Van Tulleken C. et al. (2020) HIV-1 Vpr antagonizes innate immune activation by targeting karyopherin-mediated NF-κB/IRF3 nuclear transport. eLife 9, e60821 33300875 10.7554/eLife.60821 PMC7759385

[214] Harman A.N., Nasr N., Feetham A., Galoyan A., Alshehri A.A., Rambukwelle D. et al. (2015) HIV blocks interferon induction in human dendritic cells and macrophages by dysregulation of TBK1. J. Virol. 89, 6575–6584 25855743 10.1128/JVI.00889-15 PMC4468486

[215] Saladino N., Leavitt E., Wong H.T., Ji J.H., Ebrahimi D. and Salamango D.J. (2026) DNA damage induced by HIV-1 Vpr triggers epigenetic remodeling and transcriptional programs to enhance virus transcription and latency reactivation. PLoS Biol. 24, e3003621 41628238 10.1371/journal.pbio.3003621 PMC12875578

[216] Sheehy A.M., Gaddis N.C., Choi J.D. and Malim M.H. (2002) Isolation of a human gene that inhibits HIV-1 infection and is suppressed by the viral Vif protein. Nature 418, 646–650 12167863 10.1038/nature00939

[217] Yu X., Yu Y., Liu B., Luo K., Kong W., Mao P. et al. (2003) Induction of APOBEC3G ubiquitination and degradation by an HIV-1 Vif-Cul5-SCF complex. Science 302, 1056–1060 14564014 10.1126/science.1089591

[218] Zheng Y.H., Irwin D., Kurosu T., Tokunaga K., Sata T. and Peterlin B.M. (2004) Human APOBEC3F is another host factor that blocks human immunodeficiency virus type 1 replication. J. Virol. 78, 6073–6076 15141007 10.1128/JVI.78.11.6073-6076.2004 PMC415831

[219] Liddament M.T., Brown W.L., Schumacher A.J. and Harris R.S. (2004) APOBEC3F properties and hypermutation preferences indicate activity against HIV-1 *in vivo*. Curr. Biol. 14, 1385–1391 15296757 10.1016/j.cub.2004.06.050

[220] Wiegand H.L., Doehle B.P., Bogerd H.P. and Cullen B.R. (2004) A second human antiretroviral factor, APOBEC3F, is suppressed by the HIV-1 and HIV-2 Vif proteins. EMBO J. 23, 2451–2458 15152192 10.1038/sj.emboj.7600246 PMC423288

[221] Harari A., Ooms M., Mulder L.C.F. and Simon V. (2009) Polymorphisms and splice variants influence the antiretroviral activity of human APOBEC3H. J. Virol. 83, 295–303 18945781 10.1128/JVI.01665-08 PMC2612324

[222] OhAinle M., Kerns J.A., Li M.M.H., Malik H.S. and Emerman M. (2008) Antiretroelement activity of APOBEC3H was lost twice in recent human evolution. Cell Host Microbe 4, 249–259 18779051 10.1016/j.chom.2008.07.005 PMC2608726

[223] Zhang W., Du J., Evans S.L., Yu Y. and Yu X.F. (2011) T-cell differentiation factor CBF-β regulates HIV-1 Vif-mediated evasion of host restriction. Nature 481, 376–379 22190036 10.1038/nature10718

[224] Greenwood E.J., Matheson N.J., Wals K., van den Boomen D.J., Antrobus R., Williamson J.C. et al. (2016) Temporal proteomic analysis of HIV infection reveals remodelling of the host phosphoproteome by lentiviral Vif variants. Elife 5, e18296 27690223 10.7554/eLife.18296 PMC5085607

[225] Naamati A., Williamson J.C., Greenwood E.J., Marelli S., Lehner P.J. and Matheson N.J. (2019) Functional proteomic atlas of HIV infection in primary human CD4^+^ T cells. Elife 8, e41431 30857592 10.7554/eLife.41431 PMC6414203

[226] Pan Q., Rong L., Zhao X. and Liang C. (2009) Fragile X mental retardation protein restricts replication of human immunodeficiency virus type 1. Virology 387, 127–135 19249802 10.1016/j.virol.2009.02.006

[227] Lin Z., Su X., Chen W., Ci B., Zhang S. and Lin H. (2014) Dph7 catalyzes a previously unknown demethylation step in diphthamide biosynthesis. J. Am. Chem. Soc. 136, 6179–6182 24739148 10.1021/ja5009272 PMC4015618

[228] Kelenis D.P., Johnson J.R., Sidoli S., Emery A., Swanstrom R., Hawkins L.J. et al. (2026) HIV-1 infection induces Vif-mediated SUMOylation of host RNA splicing factors important for proper viral RNA splicing. Proc. Natl. Acad. Sci. U.S.A. 123, e2532659123 42048450 10.1073/pnas.2532659123 PMC13142951

[229] Bandini M., Ghone D., Evans E.L., Suzuki A. and Sherer N.M. (2025) HIV-1 Vif and Vpr cooperatively modulate the cell cycle to maximize per-cell virion production. Proc. Natl. Acad. Sci. U.S.A. 122, e2511502122 41196352 10.1073/pnas.2511502122 PMC12626010

[230] DeHart J.L., Bosque A., Harris R.S. and Planelles V. (2008) Human immunodeficiency virus type 1 Vif induces cell cycle delay via recruitment of the same E3 ubiquitin ligase complex that targets APOBEC3 proteins for degradation. J. Virol. 82, 9265–9272 18596088 10.1128/JVI.00377-08 PMC2546900

[231] Ghone D., Evans E.L., Bandini M., Stephenson K.G., Sherer N.M. and Suzuki A. (2025) HIV-1 Vif disrupts phosphatase feedback regulation at the kinetochore, leading to a pronounced pseudo-metaphase arrest. Elife 13, RP101136 40080415 10.7554/eLife.101136 PMC11906157

[232] Du J., Rui Y., Zheng W., Li P., Kang J., Zhao K. et al. (2019) Vif-CBFβ interaction is essential for Vif-induced cell cycle arrest. Biochem. Biophys. Res. Commun. 511, 910–915 30851937 10.1016/j.bbrc.2019.02.136

[233] Marelli S., Williamson J.C., Protasio A.V., Naamati A., Greenwood E.J., Deane J.E. et al. (2020) Antagonism of PP2A is an independent and conserved function of HIV-1 Vif and causes cell cycle arrest. Elife 9, e53036 32292164 10.7554/eLife.53036 PMC7920553

[234] Nagata K., Shindo K., Matsui Y., Shirakawa K. and Takaori-Kondo A. (2020) Critical role of PP2A-B56 family protein degradation in HIV-1 Vif mediated G2 cell cycle arrest. Biochem. Biophys. Res. Commun. 527, 257–263 32446377 10.1016/j.bbrc.2020.04.123

[235] Foley E.A., Maldonado M. and Kapoor T.M. (2011) Formation of stable attachments between kinetochores and microtubules depends on the B56-PP2A phosphatase. Nat. Cell Biol. 13, 1265–1271 21874008 10.1038/ncb2327 PMC3186838

[236] Wang J., Shackelford J.M., Casella C.R., Shivers D.K., Rapaport E.L., Liu B. et al. (2007) The Vif accessory protein alters the cell cycle of human immunodeficiency virus type 1 infected cells. Virology 359, 243–252 17056089 10.1016/j.virol.2006.09.026 PMC1934563

[237] Salamango D.J., Ikeda T., Moghadasi S.A., Wang J., McCann J.L., Serebrenik A.A. et al. (2019) HIV-1 Vif triggers cell cycle arrest by degrading cellular PPP2R5 phospho-regulators. Cell Rep. 29, 1057–1065.e4 31665623 10.1016/j.celrep.2019.09.057 PMC6903395

[238] Evans E.L., Becker J.T., Fricke S.L., Patel K. and Sherer N.M. (2018) HIV-1 Vif’s capacity to manipulate the cell cycle is species specific. J. Virol. 92, e02102–17 29321323 10.1128/JVI.02102-17 PMC5972884

[239] Wang J., Reuschel E.L., Shackelford J.M., Jeang L., Shivers D.K., Diehl J.A. et al. (2011) HIV-1 Vif promotes the G_1_- to S-phase cell-cycle transition. Blood 117, 1260–1269 21149631 10.1182/blood-2010-06-289215 PMC3320848

[240] Abraham R.T. (2004) The ATM-related kinase, hSMG-1, bridges genome and RNA surveillance pathways. DNA Repair (Amst.) 3, 919–925 15279777 10.1016/j.dnarep.2004.04.003

[241] Salamango D.J., McCann J.L., Demir Ö., Becker J.T., Wang J., Lingappa J.R. et al. (2020) Functional and structural insights into a Vif/PPP2R5 complex elucidated using patient HIV-1 isolates and computational modeling. J. Virol. 94, e00631–20 32847850 10.1128/JVI.00631-20 PMC7565612

[242] Wlodarchak N. and Xing Y. (2016) PP2A as a master regulator of the cell cycle. Crit. Rev. Biochem. Mol. Biol. 51, 162–184 26906453 10.3109/10409238.2016.1143913 PMC4905575

[243] Vader G. and Lens S.M.A. (2008) The Aurora kinase family in cell division and cancer. Biochim. Biophys. Acta 1786, 60–72 18662747 10.1016/j.bbcan.2008.07.003

[244] Cimini D., Wan X., Hirel C.B. and Salmon E.D. (2006) Aurora kinase promotes turnover of kinetochore microtubules to reduce chromosome segregation errors. Curr. Biol. 16, 1711–1718 16950108 10.1016/j.cub.2006.07.022

[245] Lan W., Zhang X., Kline-Smith S.L., Rosasco S.E., Barrett-Wilt G.A., Shabanowitz J. et al. (2004) Aurora B phosphorylates centromeric MCAK and regulates its localization and microtubule depolymerization activity. Curr. Biol. 14, 273–286 14972678 10.1016/j.cub.2004.01.055

[246] Salamango D.J. and Harris R.S. (2021) Demystifying cell cycle arrest by HIV-1 Vif. Trends Microbiol. 29, 381–384 33478820 10.1016/j.tim.2021.01.001 PMC8416294

[247] Guerrero J.F., Zimdars L.L., Bruce J.W., Becker J.T., Evans E.L., Torabi S. et al. (2025) Single-cell delineation of strain-specific HIV-1 Vif activities using dual reporter sensor cells and live cell imaging. J. Virol. 99, e0157924 39998123 10.1128/jvi.01579-24 PMC11915839

[248] Binka M., Ooms M., Steward M. and Simon V. (2012) The activity spectrum of Vif from multiple HIV-1 subtypes against APOBEC3G, APOBEC3F, and APOBEC3H. J. Virol. 86, 49–59 22013041 10.1128/JVI.06082-11 PMC3255910

[249] Guerrero J.F., Lesko S.L., Evans E.L. and Sherer N.M. (2024) Studying retroviral life cycles using visible viruses and live cell imaging. Annu. Rev. Virol. 11, 125–146 38876144 10.1146/annurev-virology-100422-012608 PMC11697243

[250] Wong H.T., Luperchio A.M., Riley S. and Salamango D.J. (2023) Inhibition of ATM-directed antiviral responses by HIV-1 Vif. PLoS Pathog. 19, e1011634 37669285 10.1371/journal.ppat.1011634 PMC10503699

[251] Felzien L.K., Woffendin C., Hottiger M.O., Subbramanian R.A., Cohen E.A. and Nabel G.J. (1998) HIV transcriptional activation by the accessory protein, VPR, is mediated by the p300 co-activator. Proc. Natl. Acad. Sci. U.S.A. 95, 5281–5286 9560267 10.1073/pnas.95.9.5281 PMC20252

[252] Saladino N., Leavitt E., Wong H.T., Ji J.H., Ebrahimi D. and Salamango D.J. (2026) DNA damage induced by HIV-1 Vpr triggers epigenetic remodeling and transcriptional programs to enhance virus transcription and latency reactivation. PLoS Biol. 24, e3003621 41628238 10.1371/journal.pbio.3003621 PMC12875578

[253] Stewart S.A., Poon B., Jowett J.B. and Chen I.S. (1997) Human immunodeficiency virus type 1 Vpr induces apoptosis following cell cycle arrest. J. Virol. 71, 5579–5592 9188632 10.1128/jvi.71.7.5579-5592.1997 PMC191800

[254] Nishizawa M., Kamata M., Mojin T., Nakai Y. and Aida Y. (2000) Induction of apoptosis by the Vpr protein of human immunodeficiency virus type 1 occurs independently of G(2) arrest of the cell cycle. Virology 276, 16–26 11021990 10.1006/viro.2000.0534

[255] Ayyavoo V., Mahboubi A., Mahalingam S., Ramalingam R., Kudchodkar S., Williams W.V. et al. (1997) HIV-1 Vpr suppresses immune activation and apoptosis through regulation of nuclear factor kappa B. Nat. Med. 3, 1117–1123 9334723 10.1038/nm1097-1117

[256] Newcomb R., Dean E., McKinney B.J. and Alvarez J.V. (2021) Context-dependent effects of whole-genome duplication during mammary tumor recurrence. Sci. Rep. 11, 14932 34294755 10.1038/s41598-021-94332-z PMC8298634

[257] Schukken K.M. and Sheltzer J.M. (2022) Extensive protein dosage compensation in aneuploid human cancers. Genome Res. 32, 1254–1270 35701073 10.1101/gr.276378.121 PMC9341510

[258] Yahya G., Menges P., Amponsah P.S., Ngandiri D.A., Schulz D., Wallek A. et al. (2022) Sublinear scaling of the cellular proteome with ploidy. Nat. Commun. 13, 6182 36261409 10.1038/s41467-022-33904-7 PMC9581932

[259] Gemble S., Wardenaar R., Keuper K., Srivastava N., Nano M., Macé A.S. et al. (2022) Genetic instability from a single S phase after whole-genome duplication. Nature 604, 146–151 35355016 10.1038/s41586-022-04578-4 PMC8986533

[260] Conti L., Matarrese P., Varano B., Gauzzi M.C., Sato A., Malorni W. et al. (2000) Dual role of the HIV-1 vpr protein in the modulation of the apoptotic response of T cells. J. Immunol. 165, 3293–3300 10975846 10.4049/jimmunol.165.6.3293

[261] Okumura A., Alce T., Lubyova B., Ezelle H., Strebel K. and Pitha P.M. (2008) HIV-1 accessory proteins VPR and Vif modulate antiviral response by targeting IRF-3 for degradation. Virology 373, 85–97 18082865 10.1016/j.virol.2007.10.042 PMC2312338

[262] Johnson J.R., Crosby D.C., Hultquist J.F., Kurland A.P., Adhikary P., Li D. et al. (2022) Global post-translational modification profiling of HIV-1-infected cells reveals mechanisms of host cellular pathway remodeling. Cell Rep. 39, 110690 35417684 10.1016/j.celrep.2022.110690 PMC9429972

[263] Virgilio M.C., Ramnani B., Chen T., Disbennett W.M., Lubow J., Welch J.D. et al. (2024) HIV-1 Vpr combats the PU.1-driven antiviral response in primary human macrophages. Nat. Commun. 15, 5514 38951492 10.1038/s41467-024-49635-w PMC11217462

[264] Bressy C., Droby G.N., Maldonado B.D., Steuerwald N. and Grdzelishvili V.Z. (2019) Cell cycle arrest in G2/M phase enhances replication of interferon-sensitive cytoplasmic RNA viruses via inhibition of antiviral gene expression. J. Virol. 93, e01885–18 30487274 10.1128/JVI.01885-18 PMC6364032

[265] Mesev E.V., LeDesma R.A. and Ploss A. (2019) Decoding type I and III interferon signalling during viral infection. Nat. Microbiol. 4, 914–924 30936491 10.1038/s41564-019-0421-x PMC6554024

[266] Chan Y.K. and Gack M.U. (2016) Viral evasion of intracellular DNA and RNA sensing. Nat. Rev. Microbiol. 14, 360–373 27174148 10.1038/nrmicro.2016.45 PMC5072394

[267] Alcami A. and Koszinowski U.H. (2000) Viral mechanisms of immune evasion. Mol. Med. Today 6, 365–372 10954870 10.1016/S1357-4310(00)01775-5 PMC7141564

[268] Rice A.P. and Kimata J.T. (2015) Subversion of cell cycle regulatory mechanisms by HIV. Cell Host Microbe 17, 736–740 26067601 10.1016/j.chom.2015.05.010 PMC4476643

[269] Lewis C.A., Margolis D.M. and Browne E.P. (2025) HIV-1 accessory protein Vpr possesses a cryptic p300-dependent transcription-promoting activity that is blocked by histone deacetylases in CD4^+^ T cells. PLoS Pathog. 21, e1013073 40901928 10.1371/journal.ppat.1013073 PMC12419630

[270] Atindaana E., Kissi-Twum A., Emery S., Burnett C., Pitcher J., Visser M. et al. (2022) Bimodal expression patterns, and not viral burst sizes, predict the effects of Vpr on HIV-1 proviral populations in jurkat cells. mBio 13, e0374821 35384697 10.1128/mbio.03748-21 PMC9040753

[271] Reuschl A.K., Mesner D., Shivkumar M., Whelan M.V.X., Pallett L.J., Guerra-Assunção J.A. et al. (2022) HIV-1 Vpr drives a tissue residency-like phenotype during selective infection of resting memory T cells. Cell Rep. 39, 110650 35417711 10.1016/j.celrep.2022.110650 PMC9350556

[272] Hrimech M., Yao X.J., Bachand F., Rougeau N. and Cohen É.A. (1999) Human immunodeficiency virus type 1 (HIV-1) Vpr functions as an immediate-early protein during HIV-1 infection. J. Virol. 73, 4101–4109 10196306 10.1128/JVI.73.5.4101-4109.1999 PMC104189

[273] Gummuluru S. and Emerman M. (1999) Cell cycle- and Vpr-mediated regulation of human immunodeficiency virus type 1 expression in primary and transformed T-cell lines. J. Virol. 73, 5422–5430 10364289 10.1128/JVI.73.7.5422-5430.1999 PMC112598

[274] Deeks S.G. (2012) Shock and kill. Nature 487, 439–440 22836995 10.1038/487439a

[275] Darcis G., Van Driessche B. and Van Lint C. (2017) HIV latency: should we shock or lock? Trends Immunol. 38, 217–228 28073694 10.1016/j.it.2016.12.003

[276] Zhou D., Hayashi T., Jean M., Kong W., Fiches G., Biswas A. et al. (2020) Inhibition of Polo-like kinase 1 (PLK1) facilitates the elimination of HIV-1 viral reservoirs in CD4^+^ T cells *ex vivo*. Sci. Adv. 6, eaba1941 32832623 10.1126/sciadv.aba1941 PMC7439358

[277] Garcia-Vidal E., Badia R., Pujantell M., Castellví M., Felip E., Clotet B. et al. (2019) Dual effect of the broad spectrum kinase inhibitor midostaurin in acute and latent HIV-1 infection. Antiviral Res. 168, 18–27 31077767 10.1016/j.antiviral.2019.05.003

[278] Yang H.C., Xing S., Shan L., O'Connell K., Dinoso J., Shen A. et al. (2009) Small-molecule screening using a human primary cell model of HIV latency identifies compounds that reverse latency without cellular activation. J. Clin. Invest. 119, 3473–3486 19805909 10.1172/JCI39199 PMC2769176

[279] Rice A.P. (2016) Cyclin-dependent kinases as therapeutic targets for HIV-1 infection. Expert Opin. Ther. Targets 20, 1453–1461 27797603 10.1080/14728222.2016.1254619 PMC5219930

